# Long-term field comparison of multiple low-cost particulate matter sensors in an outdoor urban environment

**DOI:** 10.1038/s41598-019-43716-3

**Published:** 2019-05-16

**Authors:** Florentin M. J. Bulot, Steven J. Johnston, Philip J. Basford, Natasha H. C. Easton, Mihaela Apetroaie-Cristea, Gavin L. Foster, Andrew K. R. Morris, Simon J. Cox, Matthew Loxham

**Affiliations:** 1 Faculty of Engineering and Physical Sciences, University of Southampton, Southampton, UK; 2 Southampton Marine and Maritime Institute, University of Southampton, Southampton, UK; 3 Faculty of Environmental and Life Sciences, University of Southampton, Southampton, UK; 4 School of Ocean and Earth Science, National Oceanography Centre, University of Southampton, Southampton, UK; 5 National Oceanography Centre, Southampton, UK; 6 Faculty of Medicine, University of Southampton, Southampton, UK; 7 National Institute for Health Research, Southampton Biomedical Research Centre, Southampton, UK; 8 Institute for Life Sciences, University of Southampton, Southampton, UK

**Keywords:** Environmental sciences, Atmospheric science

## Abstract

Exposure to ambient particulate matter (PM) air pollution is a leading risk factor for morbidity and mortality, associated with up to 8.9 million deaths/year worldwide. Measurement of personal exposure to PM is hindered by poor spatial resolution of monitoring networks. Low-cost PM sensors may improve monitoring resolution in a cost-effective manner but there are doubts regarding data reliability. PM sensor boxes were constructed using four low-cost PM micro-sensor models. Three boxes were deployed at each of two schools in Southampton, UK, for around one year and sensor performance was analysed. Comparison of sensor readings with a nearby background station showed moderate to good correlation (0.61 < r < 0.88, p < 0.0001), but indicated that low-cost sensor performance varies with different PM sources and background concentrations, and to a lesser extent relative humidity and temperature. This may have implications for their potential use in different locations. Data also indicates that these sensors can track short-lived events of pollution, especially in conjunction with wind data. We conclude that, with appropriate consideration of potential confounding factors, low-cost PM sensors may be suitable for PM monitoring where reference-standard equipment is not available or feasible, and that they may be useful in studying spatially localised airborne PM concentrations.

## Introduction

Exposure to particulate matter (PM) air pollution is one of the leading causes of morbidity and mortality globally, being responsible for between 4.2 million to 8.9 million deaths per year worldwide^1–4^. PM exposure is associated with increased risk of lung cancer, asthma, ischaemic heart disease and strokes, while there is growing evidence for associations with chronic obstructive pulmonary disease (COPD), type 2 diabetes mellitus, and dementia^5^. PM is classified by its mean aerodynamic diameter, into size bins, usually of <10 μm (PM_10_), <2.5 μm (PM_2.5_) and <0.1 μm PM_0.1_), the latter two referred to as fine and ultrafine PM, respectively. At a diameter <10 μm, PM can be inhaled into the respiratory tract. Fine PM is respirable and can reach bronchioles and potentially alveoli, while ultrafine PM is able to cross the air-blood barrier and enter the circulation^6,7^.

PM originates from a variety of sources - a recent global review of source apportionment studies, estimated that 25% of PM_2.5_ pollution originates from traffic, 22% from unspecified sources of human origins, 20% from domestic fuel burning, 18% from natural dust and salt and 15% from industrial activities^8^. Within urban areas, relative and total contribution of these sources varies spatially and temporally, being further modulated by weather conditions^9^. While reference-level monitoring stations can capture temporal fluctuations in PM concentration, their cost, expertise for maintenance and size mean it is not feasible to use them to obtain the spatial granularity required to understand spatially heterogeneous PM concentrations across urban environments strongly influenced by localised sources of pollution^10–12^.

The most common reference techniques for monitoring PM are the Tapered Element Oscillating Microbalance (TEOM) and Beta Attenuation Monitor (BAM), both of which measure properties of PM directly related to PM mass. Commercially available low-cost PM sensors may provide an opportunity to complement the existing monitoring network^13^. Unlike reference-grade instruments, these PM sensors generally utilise light-scattering and can detect particles with aerodynamic diameters 0.3–10 μm. Particles with diameter <0.3 μm do not scatter light sufficiently, while PM >10 μm are not easy to draw into the sensor^14^. Since such sensors work by detecting particles by number, they are only able to infer PM mass.

These sensors are more suitable for deployment in large numbers in terms of their cost but questions remain regarding the quality of data produced^15–17^ and their precision and accuracy may not be sufficient for regulatory use^18^. Various models of low-cost PM sensors have been tested both under controlled conditions and in the field but the validity and variability of the data they produce is poorly understood^19^. The available evidence suggests PM concentrations reported by low-cost PM sensors are of questionable accuracy^20^, with their reliability varying depending on PM concentration^20–22^ and meteorological factors^23^ such as relative humidity^24,25^. This has led to suggestions that each low-cost PM sensor should be tested under conditions as close as possible to those at its potential deployment site^19^. Moreover, low-cost PM sensors are strongly influenced by aerosol composition^19^ and it is thus necessary to study them in different environments. The low-cost PM sensors selected here (see Table 1) have been studied before^21,23–33^ (with the exception of the Honeywell HPMA115S0). Such studies generally analysed the data produced by the sensors over short period of time (a few days to some weeks)^21,23–28,30,33^. Sayahi *et al*.^32^ compared two Plantower PMS1003 and two Plantower PMS5003 in the field for 320 days, over two different periods of time, comparing hourly and daily readings to a collocated TEOM; Badura *et al*.^29^ compared a total of 12 sensors from four different manufacturers (three of each) including the Plantower PMS7003 and the Alphasense OPC-N2 against a TEOM for half a year with four different time average period ranging from 1 min to 1 h; Feinberg *et al*.^31^ compared a total of 21 low-cost PM sensors from seven different manufacturers (three of each) including the Alphasense OPC-N2 for seven months against a GRIMM EDM 180 dust monitor for 1 h, 12 h and 24 h data.

**Table 1:** Main characteristics of the fan assisted low-cost PM sensors used in the study. Adapted^34,35^. Prices accurate at the time of construction.

Model	Size (mm) (H × W × D)	Price (USD)	Detection range (μm)	Concentration range (μg/m^3^)	Declared Accuracy (μg/m^3^)	Sampling interval (s)	Particle count
Alphasense OPC-N2	60 × 64 × 75	443	0.38 to 17	0.01 to 1,500	Not known	1 to 10	Yes
Plantower PMS5003	38 × 21 × 50	28	0.3 to 10	0 to 500	±10	1	Yes
Plantower PMS7003	37 × 12 × 48	28	0.3 to 10	0 to 500	±10	1	Yes
Honeywell HPMA115S0	36 × 43 × 24	33	Not known	0 to 1,000	±15	<6	No

In this study, we have characterised the field-based performance of different PM sensors at two school sites in Southampton, UK, over ≈a year. The aim of this study was the long-term evaluation of: (1) the capacity of the sensors to produce hourly data, informing about trends in pollution, with correlation with reference instruments; (2) the need to host several low-cost sensors at the same place; (3) the usefulness of the sensors to produce spatial information about air pollution; (4) the capacity of these sensors to detect short-lived event not detected by Automatic Urban and Rural Network (AURN) stations. We also provide some recommendations and best practices about the use of these sensors in the context of real-world monitoring situation. To our knowledge, this is the first study to collocate multiple sensors of different models in a field setting configured as a network of sensors for an extended duration (≈a year long) and compare them against each other as well as against reference instruments. This allows us to determine the effects of external factors such as background pollution and meteorological conditions on sensor performance over a full year of environmental conditions, as well as studying their response to a range of short-lived events of pollution. It is also the first to conduct a long-term sensor evaluation of these sensors in a coastal port city.

## Methods

### Area of the study

In February 2018, we deployed a network of low-cost sensors to monitor PM concentrations at two schools in Southampton, UK^34,35^. The data analysed in this study covers the period from 13/03/18 until 28/02/19, the full datasets used are available in the Additional Information. Southampton is located on the south coast of England with population ≈236900 and area ≈52 km^2^. The city is surrounded on three sides by motorways (M3, M27, M271). In the south of the city is the busiest cruise port in Europe and one of the largest ports in the UK^36^, leading to Southampton water and the Solent, a strait of the English Channel. A passenger airport is located 5 km NNE of the city centre. Southampton has been identified by the UK Government as one of a number of cities which need to improve air quality.

### PM_2.5_ monitoring in Southampton

In Southampton, PM_2.5_ is monitored by a single AURN background station located in the city centre^37^ with a roadside AURN station (A33 station) monitoring PM_10_ and nitrogen oxides. The next nearest AURN station monitoring PM_2.5_ is located in Portsmouth, 40 km to the East. The background station is equipped with a FDMS 8500 and a TEOM 1400ab Ambient Particulate Monitor which report the PM_2.5_ concentration levels hourly along with the volatile and non-volatile PM_2.5_. The inlet is situated on the roof of the AURN station at 4 m above ground level. The FDMS TEOM reads concentration changes on a 12 min cycle. Hourly averages are available at http://www.airquality.co.uk.

### Meteorological conditions and PM_2.5_ during the study

Supplementary Fig. S2 shows the daily mean of temperature, humidity and wind direction and speed over the study and PM_2.5_ concentrations recorded by the AURN station. The meteorological data presented here was recorded by Southampton Weather station and the data is readily available from http://www.southamptonweather.co.uk/. This weather station is located in the city centre (latitude: 50.899,7°; longitude: −1.395,5°). Mean air temperature was 8.8 °C (range −6.3 °C–32.1 °C) and mean relative humidity 76.4% (range 23–98%). Wind was predominantly from North-East or South-West with fewer occurrences of wind events coming from the South-East. Supplementary Fig. S1 presents the wind roses per month during the study.

### Low-cost sensor selection

Four PM sensors were compared: Plantower PMS5003^38^, Plantower PMS7003^39^, Honeywell HPMA115S0^40^ and Alphasense OPC-N2^41^. Table 1 lists their main characteristics. These sensors are controlled remotely through a Raspberry Pi and they are sufficiently small to be deployed in an enclosure small enough for mobile or wearable applications. They all report PM_2.5_ and PM_10_ concentrations in μg/m^3^. The Plantower PMS5003, the Plantower PMS7003 and the Alphasense OPC-N2 also report PM_1_ and particle count for different bin sizes - the Plantowers report size distribution for 0.3, 0.5, 1.0, 2.5, 5.0, 10 μm bins and the Alphasense OPC-N2 reports 16 bins ranging from 0.38 μm and17 μm. The Plantower sensors claim a counting efficiency of 98% for particles of diameter 0.5 μm and 50% for diameter 0.3 μm^38^. All use a sampling interval <10 s. According to the manufacturers, their accuracy is between ±10–15 μg/m^3^.

### Deployment

**Figure 1:**
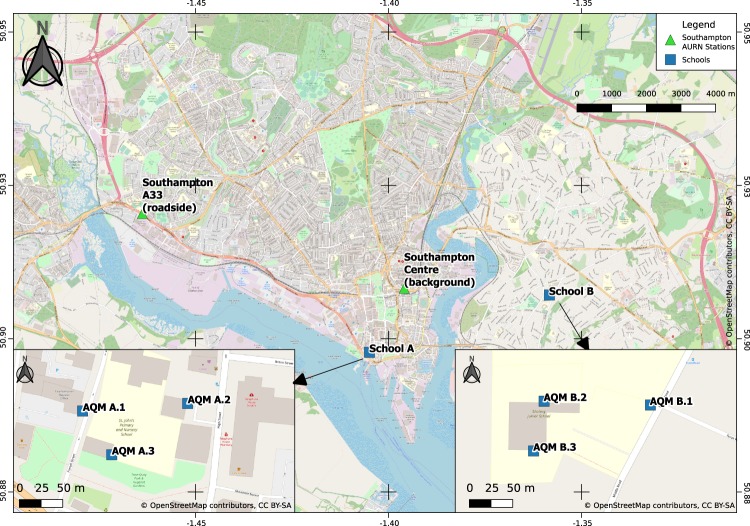
Locations of the Air Quality Monitors (AQM) deployed at School A and School B. To be noted that the vignette and the main maps have different scales. background map adapted from OpenStreetMap^53^. The cartography in the OpenStreetMap map tiles is licensed under CC BY-SA (www.openstreetmap.org/copyright). The licence terms can be found on the following link: http://creativecommons.org/licenses/by-sa/2.0/.

The sensors were assembled into an Air Quality Monitor (AQM) as described previously^34,35^. A brief description of the AQMs is available in Supplementary Section 1. AQMs were deployed outdoor in two schools in distinct areas of Southampton from February 2018. The two schools were selected as part of a pilot project by Southampton City Council and had the added interest of monitoring PM exposure of a vulnerable population^5^. School A is located 1.3 km SW of the background AURN station and School B is located 2.7 km E of the AURN station. For each school (Fig. 1), one AQM was sited <10 m away from the school driveway to record exposure near road PM (AQM A.1 and AQM B.1) while the two others were located further from the road (≈40 m for School A and ≈100 m for School B), in the playground (AQM A.3 and AQM B.2) with the third one placed sited to obtain a good coverage of the school away from the two other monitors. The height of the AQM (<2 m below AURN inlet, see Table 2) was imposed by physical constraints (access to power supply, protection against vandalism, protection against ball games in the playground, safety). In each case, the AQM have been placed as close to the ground as possible.

**Table 2:** Individual sensors uptime per location and comparison with the reference background station. For AQM B.1 the Alphasense OPC-N2 reported a random signal. The Honeywell HPMA115S0 was only operational for AQM A.1. The table also presents some characteristics of the different sites.

		Honeywell HPMA115S0	Plantower PMS5003	Plantower PMS7003	Alphasense OPCN2	Height above ground (m)	Location
AQM A	1	91%	94.5%	94.7%	92.6%	3.8	School entrance, East facing
	2	—	96.6%	96.6%	95.9%	2.4	School employees car park (6 parking spaces), East facing
	3	—	99.5%	52.1%	98.9%	3.5	School playground, East facing
AQM B	1	—	57.4%	99.7%	0%	2.1	School entrance, South facing
	2	—	98.9%	98.9%	56.6%	3.4	School playground, North facing
	3	—	95.7%	96.5%	95.7%	3.4	Opposite from the playground, South facing
Reference station			92.8%			4	

### Data analysis methods

Sensor performance was assessed by determination of the Pearson coefficient (r), the Spearman coefficient (*ρ*), the slope, the Root Mean Square Error (RMSE) and the Coefficient of Variation (CV). The slope is reported with a confidence interval of two standard errors, computed using a linear model based on total least square which is a non-symmetric optimisation algorithm hence the slope is non-symmetrical. RMSE characterises error against other readings, and for two measurements is calculated as $RMSE=\sqrt{mean({(X-Y)}^{2})}$. We also calculated the CV each hour for each sensor defined by $CV=\frac{\sigma }{\mu }$ where *σ* is the standard deviation of the sensor during the hour considered and *μ* is the average of the concentration of PM_2.5_ of the three sensors of the same model on the same site, providing a measure of the variability between sensors and is a measure of the precision of individual sensors^42^. A CV of zero represents a perfect agreement between sensors and a CV of 0.1 is judged to be sufficient for monitoring PM concentrations^43^. Statistical tests were conducted using a Shapiro-Wilk test of normality and a Friedman Analysis of Variance (ANOVA) followed by a Dunn post-hoc test. Data were analysed using Prism 8 (GraphPad Software, San Diego, USA) and R 3.5.1 using the packages ‘openair’, ‘lattice’, ‘dplyr’, ‘psych’ and ‘MASS’.

The sensors sampled with different time periods (between 1–6 s depending on the model) and it was necessary to take the median readings obtained over a period of time greater than the sampling period of each sensor. With short time windows, a high variability was observed for each sensors. Wider averaging windows reduce the noise of the measurements. Timeseries comparing different averaging windows are presented in Supplementary Fig. S3 for each sensors. To reduce the noise while preserving a high degree of temporal resolution the best averaging windows appears to be between 1–5 min. One of the goals of this study is to compare the behaviour of these sensors while monitoring short-lived events such as fires in the city or local sources of pollution such as the 15 min during which parents come to drop-off or pick-up their children from school. A 5 min averaging window would only provide 3 measurements for this period of time, compared to 15 measurements with 1 min averaging period. As such, it was decided that the averaging window should not exceed 1 min.

### Data quality and outlier detection

Traditional methods to detect and remove outliers use standard deviation or median absolute deviation^44^. We tested these methods with different time windows (10 min, 15 min, 30 min, 1 h, 8 h). However, we found negligible differences in correlation with the reference stations when applying these methods (Δr < 0.01) and given the currently limited understanding of the data produced by the sensors it was decided not to use these techniques.

**Figure 2:**
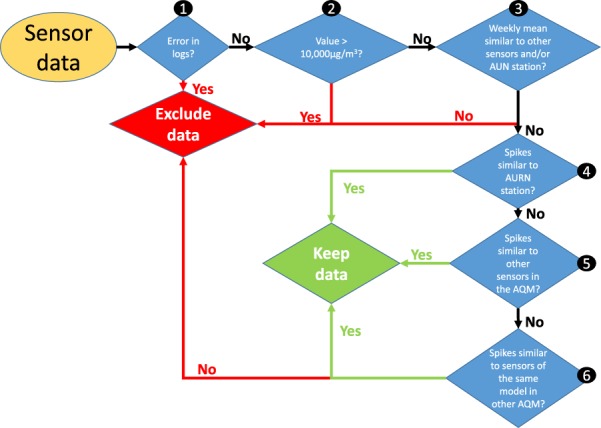
Flow chart presenting the data quality check process applied to the data from each sensor: (1) verify the logs of the sensors for error messages, (2) remove the values that are >10,000 μg/m^3^, (3) compare the weekly mean of the sensors, (4) compare the readings of the sensor to the reference station week by week to detect peaks that are present on the sensor but not on the AURN station, (5) compare the sensor with the other sensors week by week in the same Air Quality Monitor (AQM), (6) compare the sensor with the sensors of the same model week by week in the other AQM. Categories 1 and 2 are considered as time when the sensor was not operational.

To determine whether a transient signal was reliable, it was required that any peak needed to be registered by the reference station or by multiple sensors in the same AQM or in another AQM. For each sensor, the data was visualised week by week, and processed according to a non-automated algorithm presented in Fig. 2: (1) verify the logs of the sensors for error messages, (2) remove the values that are >10,000 μg/m^3^, (3) compare the weekly mean of the sensors, (4) compare the readings of the sensor to the reference station week by week to detect peaks that are present on the sensor but not on the AURN station, (5) compare the sensor with the other sensors week by week in the same Air Quality Monitor (AQM), (6) compare the sensor with the sensors of the same model week by week in the other AQM. The six steps of the process define six categories of data. Categories 1 and 2 are considered as time when the sensor was not operational. Category 3, combined with a visual inspection, enabled detection of invalid readings from the Plantower PMS7003 in AQM A.3 between 19/09/18 and 03/10/18, at which point the sensor was reset and gave reliable measurements again. It also enabled detection of a fault in the Plantower PMS5003 of AQM B.1 from the 06/10/18 which could not be resolved. The mean of the sensors during these periods are presented in Supplementary Table S1. All the other peaks identified in the dataset fell into category 4, 5 or 6 and as such were not removed from the analysis. Supplementary Figs S4, S5 and S6 present an example for each category.

The majority of the peaks observed in the dataset on multiple sensors are hypothesised to result from large fires in the city or spatially localised sources of pollution in the vicinity of the AQM such as barbecues or car emissions. Supplementary Fig. S7 describes peaks of pollution recorded by one of the AQM during a fire in the city and Supplementary Figs S13 and S14 present other peaks recorded at School B which are discussed further in the study.

## Results

Supplementary Figs S8 andS9 show the time series of PM_2.5_ concentration reported by the sensors at each location averaged by hour over the study period and compare them with the reference station. For both sites, the sensors follow similar trends to the reference station suggesting that there is similarity between PM_2.5_ concentrations at each site.

### Sensor operating period

Sensor uptime was generally high but due to technical issues, not all of the sensors were operational during the whole study (Table 2). An error occured configuring the Honeywell HPMA115S0 (a 5 V logic was used instead of a 3.3 V logic) which could only be fixed for AQM A.1 due to limited resources and access restrictions. The Alphasense OPC-N2 performed correctly in four out of six AQM. For AQM B.2, the Alphasense OPC-N2 experienced intermittent communication issues falling into categories 1 and 2 of the data quality check process from 13/03/18 until 21/06/18 and from 13/09/18 until 19/11/18 (average 2,000 error messages/day). For AQM B.1 the Alphasense OPC-N2 had unresolvable communication problems. The data produced by these sensors was discarded from this analysis. The two Plantower models performed correctly in each AQM except for Plantower PMS7003 AQM A.3 which did not perform from February to March 2018 and for the period of time detailed in the ‘Data quality and outliers detection’ section. The Honeywell HPMA115S0 stopped reporting correct data on 23/02/19 reporting a constant PM_2.5_ concentration of 1 μg/m^3^. The AURN station was not operational from the 12/07/18 until 27/07/18 and from 08/08/18 until 13/08/18. The background reference station had 92.8% uptime with downtime due to maintenance shut downs.

### Performances of the sensors between the different air quality monitors

**Figure 3:**
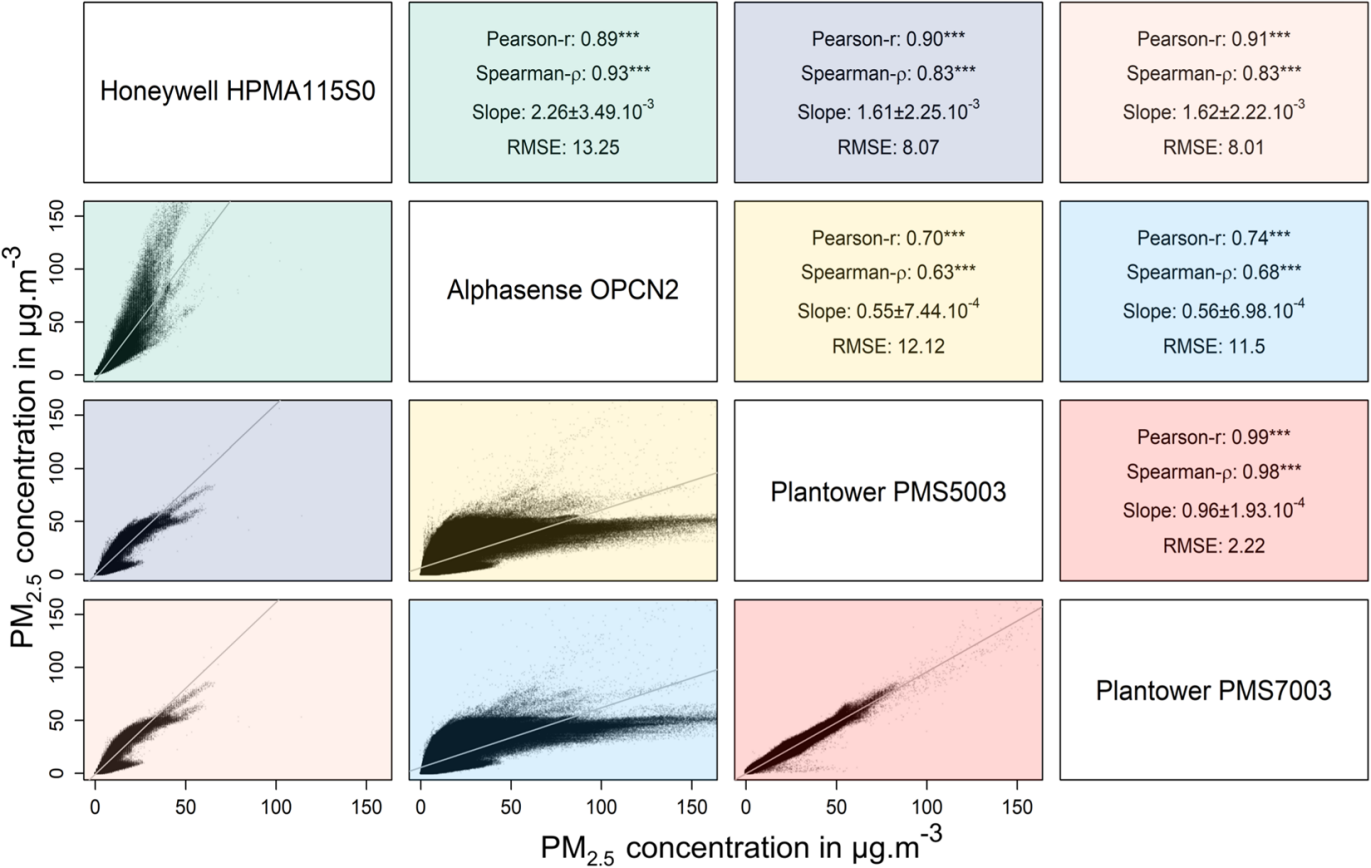
Correlation of the concentration of PM_2.5_ in μg/m^3^ reported by different PM sensor models. Graphs show reported PM_2.5_ concentrations from Plantower PMS7003 (n = 5,906,616), Plantower PMS5003 (n = 5,523,274), Alphasense OPC-N2 (n = 4,420,042) and Honeywell HPMA115S0 (n = 946,372), with the data combined from each model at each location of the study except for Honeywell HPMA115S0 (only one location available). The x-axis corresponds to the sensor named above the graph and the y-axis correspond to the sensor named to the right of the graph. The upper 0.0001% of the datapoints are not displayed. Slope is reported ±2 standard error (***p < 2 × 10^−16^).

Figure 3 shows the correlation between the reported PM_2.5_ concentrations for the different sensor models, with the data merged across the six AQMs. The Plantower PMS5003 and Plantower PMS7003 reported very high correlations (Pearson and Spearman), slope close to 1 and a RMSE of 2.22 μg/m^3^. The Alphasense OPC-N2 shows a different behaviour vs. the Plantower PMS5003 and Plantower PMS7003 with a high correlation for Pearson and a moderate correlation for Spearman, higher RMSE <12.1 μg/m^3^ and over-reporting by a factor of 2 compared to the Plantower models.

The Alphasense OPC-N2 plots present three modes - one reporting higher values than the other three sensors, one reporting similar values and one reporting lower values, the latter having fewer datapoints. This behaviour can also been observed for each AQM on Supplementary Fig. S10, apart for AQM B.2. Supplementary Fig. S11 presents the ratio of the PM_2.5_ concentrations reported by the sensors with relative humidity divided into 20 quantiles. The Alphasense OPC-N2 reports higher values than the Plantower PMS5003 for relative humidity >83%, while reporting lower values at lower relative humidity. This behaviour may therefore at least partly explain the three aforementioned modes.

The comparison of the Plantower PMS5003 and the Honeywell HPMA115S0 with relative humidity does not reveal a clear effect of relative humidity on the values reported across the two sensors, although it should be noted they correlated more at higher relative humidity potentially explaining the two trends observed between the two sensors.

The analysis of the CV between sensors of the same model within each site is presented in Table 3. For the Alphasense OPC-N2, the data is only available for School A as only one sensor of this model functioned correctly in School B. The Alphasense OPC-N2 displayed a CV of 0.11–0.17 with an upper 95^th^ percentile 0.24–0.35. The Plantower PMS5003 and the Plantower PMS7003 obtained higher values with similar results over the two locations. The Plantower PMS5003 showed a CV of 0.21–0.26 with an upper 95^th^ percentile 0.57–0.76. The Plantower PMS7003 obtained similar results. The intra-class correlation (ICC) applied to the CV with the model of sensors as subjects and the sites as raters for two-way mixed effect, consistency, single raters (ICC(3,1))^45^ yields a value of 0.94 with a p-value of 2.18 × 10^−7^ and 95^th^ confidence interval of 0.75–0.999 indicating excellent reliability of the CV and a low intra-model variability.

**Table 3:** Mean, median and 95^th^ percentile of the Coefficient of Variation (CV) per sensor and per AQM from 13/03/18 to 09/03/19. The Plantower PMS5003 in School B.1 stopped working after 06/10/18, calculations for this sensor at School B only include the period from 13/03/18 to 06/10/18.

Sensors	AQM	CV		
		Mean	Median	95^th^ percentile
Alphasense OPC-N2	A.1	0.17	0.14	0.35
	A.2	0.13	0.10	0.30
	A.3	0.11	0.08	0.24
Plantower PMS5003	A.1	0.25	0.17	0.70
	A.2	0.26	0.16	0.76
	A.3	0.21	0.14	0.57
	B.1	0.23	0.16	0.59
	B.2	0.24	0.14	0.57
	B.3	0.23	0.15	0.59
Plantower PMS7003	A.1	0.24	0.16	0.63
	A.2	0.24	0.16	0.66
	A.3	0.22	0.15	0.59
	B.1	0.25	0.18	0.67
	B.2	0.23	0.15	0.60
	B.3	0.25	0.17	0.66

Supplementary Fig. S12 presents the evolution of the CV and the concentration measured for each sensors in School A during the period ranging from 14/03/18 to 31/05/18. The CV increased as the PM concentration measured by the sensors decreased suggesting a better agreement between sensors when PM concentrations are higher.

### Correlation with the background AURN reference station

**Figure 4:**
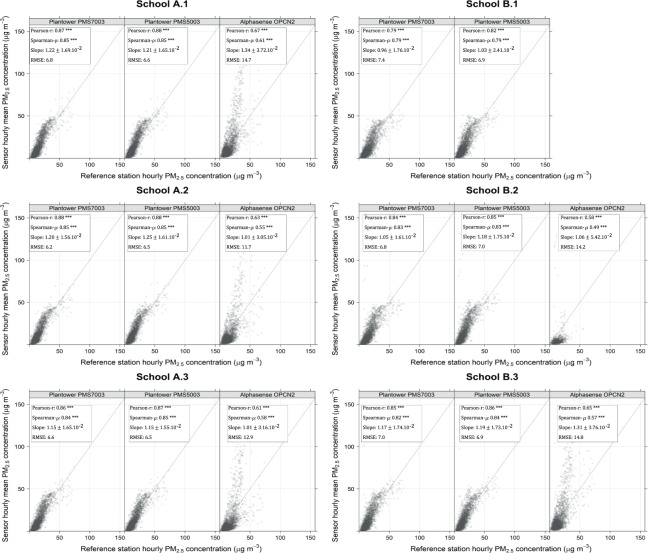
Correlation between PM_2.5_ concentrations reported by low-cost PM sensors and data from background reference station. Graphs show comparison of the PM_2.5_ concentration from Plantower PMS7003 (n = 49,255), Plantower PMS5003 (n = 46,066) and Alphasense OPC-N2 (n = 36,870), with the data combined from each model at each location of the study against the background Automatic Urban and Rural Network (AURN) station except for the Honeywell HPMA115S0 (only one location available). The upper 0.000005% of the datapoints is not displayed. The slope is given ±2 standard error (***p-value < 2 × 10^−16^).

After comparing inter-sensor performances, we studied their performance against the AURN background reference station. To study the correlation of individual sensors with the background AURN reference station, the data from each sensor was averaged to give the mean PM_2.5_ concentration per hour to match the frequency reported by the background station. Figure 4 shows the readings per AQM compared to the readings from the reference station. At School A, the Plantower PMS7003 demonstrated statistically significant high positive correlations, slopes between (1.15 ± 1.65) × 10^−2^ and (1.22 ± 1.69) × 10^−2^ and RMSE <6.8 μg/m^3^]. The Plantower PMS5003 records very similar values across the three locations of School A. The Alphasense OPC-N2 presents more variability across the three locations of School A with moderate positive correlations, slopes between (1.01 ± 3.16) × 10^−2^ and (1.34 ± 3.72) × 10^−2^ and RMSE <14.7 μg/m^3^.

In School B, the two Plantower models had very similar coefficients to School A for AQM B.2 and B.3 but the correlation coefficients were lower for AQM B.1 (r = 0.79/0.82 and *ρ* = 0.79/0.82) with a slope close to 1. For AQM B.2, the Plantower PMS5003 presents a lower slope than the Plantower PMS7003 which can be attributed to a variability in the calibration of these sensors, further illustrated by the slope obtained in Supplementary Fig. S10. For this AQM, Alphasense OPC-N2 also presents lower results than in the other locations (which performed 56.6% of the time at this location). AQM B.2 is also the AQM reporting the highest concentrations with >10 peaks during the month of August - these peaks may have resulted from barbecues nearby or wood burning. Some of these peaks were detected with a short time delay by the two other AQM of the School and mainly by AQM B.1 (a building separated AQM B.2 and AQM B.3) as presented in Supplementary Figs S13 and S14.

### Factors impacting the correlation with the background reference station

To evaluate the impact of external factors, we compared the Pearson coefficient for each sensor against the reference station, with variations in (1) month, (2) quartile of background PM_2.5_ (PM_2.5_) concentration, (3) quartile of relative humidity, (4) wind direction and (5) quartile of temperature.

**Figure 5:**
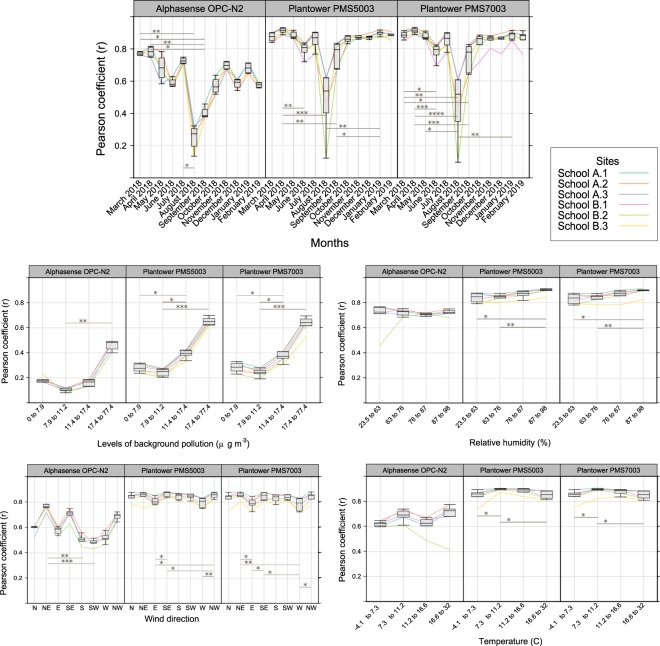
Effect of pollution and climate factors on correlation between readings from low-cost sensors and AURN background station. Graphs show variation in Pearson coefficient between the three sensor models and the background AURN reference station per site with (1) months (excluding the Alphasense OPC-N2 for AQM B.2 due to the lack of data), (2) quartile of background PM_2.5_ concentration (μg/m^3^), (3) quartile of relative humidity (RH), (4) wind direction and (5) quartile of temperature. August has been excluded from the graphs except for (1). Each box represents median, the 25^th^ and 75^th^ percentiles with maximum and minimum whiskers of Pearson coefficient for the locations considered (n = 4 for the Alphasense OPC-N2 and n = 6 the Plantowers). Data analysed using a Friedman analysis of variance (ANOVA) with Dunn’s post-hoc test for pairwise comparison, *p < 0.05, **p < 0.01, ***p < 0.001, ****p < 0.0001.

The Shapiro-Wilk tests of normality conducted on the different subsets of data revealed that some groups of Pearson coefficients were not normally distributed. To verify the statistical significance observed between the different categories, we conducted a non-parametric statistical analysis on the groups of data with a Friedman ANOVA with Dunn multiple comparison test to determine pairwise comparisons driving the differences. The analysis was not conducted on the Honeywell HPMA115S0 due to the paucity of sites. Figure 5 shows the Box and Whisker plots of the Pearson coefficients for the different sensors at the different sites with the five potential confounding factors. The graphs for relative humidity, background pollution, wind direction and temperature does not include the data collected in August (see ‘Month’ and ‘Combined effects’ below). The analysis including August is presented in Supplementary Fig. S15.

#### Month

The Alphasense OPC-N2 for AQM B.2 has not been included in this analysis due to the lack of data for this sensor. The significant differences observed are mostly involving August, driven down by the AQM located in School B and in particular by AQM B.2 for which the three sensors performing registered notable peak of pollution, recorded to a lesser magnitude by AQM B.1 and AQM B.3 as presented in Supplementary Fig. S13 and with a zoom on one specific spike in Supplementary Fig. S14 where we can see the spike monitored successively by AQM B.2, then AQM B.3 and finally AQM B.2 with a 1 min interval between each location wind speed being <0.8 m/s. These peaks were not recorded by the reference station or by the AQMs in School A. Given that these readings are present on the three AQM at School B, they may indicate a very localised source of pollution illustrating the capacity of these sensors to detect highly localised sources of pollution. To remove the effect observed during August on the analysis of the other environmental factors, the data produced during this month has been excluded from the analysis. The two Plantowers do not show a drop in performances but the Alphasense OPC-N2 appears to show a trend for decreasing correlation with months although no significant differences could be observed between the beginning and the end of the study.

#### Background concentration

All sensors showed increasing correlation with the background station as background pollution increased with Pearson coefficients >0.6 for the Plantowers and >0.4 for the Alphasense OPC-N2 for background pollution in the upper quartile (17.8 to 77.4 μg/m^3^). This value fell with decreasing pollution for all sensors, but the drop was more pronounced for the Alphasense OPC-N2, whereas the lowest Pearson coefficient was at a background concentration in the second quartile (8.2–11.4 μg/m^3^, p < 0.01) vs. upper quartile. For the 3 sensors, the second background PM_2.5_ quartile showed a significantly lower Pearson correlation than the highest group.

#### Relative humidity

The two Plantower sensors presented a better correlation with the background PM_2.5_ station at the upper quartiles of relative humidity (76–98% RH). For the Alphasense OPC-N2, there was no significant difference between the different quartiles while the correlation dropped for the third quartile. Supplementary Fig. S16 presents the same analysis conducted using ventiles of relative humidity showing that the correlation increases with relative humidity up to the last ventile (96–98%). At lower relative humidity there is also more variability across the sites illustrated by the spread of the boxes with the Alphasense OPC-N2 presenting the widest range of values. The two Plantower sensors present significant differences between their upper quartile and their first and second quartiles.

#### Wind direction

The two Plantower sensors present little variability with wind direction. The Alphasense OPC-N2 has lower correlation coefficients for wind from S, SW and W. All of the significant differences observed involve at least one of these directions and are more pronounced for the sensors located in School B. The differences observed are likely to result from the combined effect of environmental factors as these three directions are the one for which the lower background concentration were registered by the reference station with W being the direction recording the lowest background concentration. These directions are from the coast and are likely subject to fewer sources of pollution than directions coming from inland.

#### Temperature

The correlation between AQM sensors and background station readings was higher at temperatures in the second quartile (7.3–11.2 °C). The Alphasense OPC-N2 showed a drop in correlation for the first and third quartile and shows more variability with temperature compared to the Plantower models. Conversely, the Plantower models show significant differences between their quartiles but with little variation of correlation.

#### Combined effects

A linear model predicting background concentration with month, wind direction, relative humidity and temperature gave a statistically significant adjusted R^2^ of 0.11. A linear model including only wind direction and months also obtained a statistically significant adjusted *R*^2^ of 0.11 (adjusted *R*^2^ of 0.07 when only taking into account months only) and a linear model including only relative humidity and temperature obtained a statistically significant adjusted *R*^2^ of 0.001 suggesting a strong effect of months on background concentration and a more limited effect wind direction. The lowest correlations were during August. The analysis of the wind direction during this month showed that the wind was blowing mostly from SW which is also the direction for which we observed the lowest correlation between AQM sensor and background reference station readings. This difference is further illustrated by the differences observed between Fig. 5 and Supplementary Fig. S15 which suggest a confounding source of pollution from SW in August. Two possible explanations for this are local combustion events in particular barbecues, burning of garden waste or PM generating works taking at the school during the summer holiday. Supplementary Figs S13 and S14 focus on these period of time and support the hypothesis of burning of garden waste or barbecues given the times when these events happens after 7 pm during a particularly warm August.

## Discussion

We have evaluated the performance of four models of low-cost PM sensor characterising inter-model performance as well as performance against the nearest reference station, examining potential confounding conditions. The sensors generally operated reliably with 14 sensors out of 19 (five sensors being misconfigured) having uptime greater than the reference station. The sensors showed medium to high correlation between each other while performing differently with variable relative humidity. The correlation of the sensors with the reference station is strongly influenced by the background concentration and drift over-time was observed for the Alphasense OPC-N2. Relative humidity and temperature have a limited influence in general, albeit statistically significant for the Plantowers. Month and wind direction have a combined effect on the correlation suggesting different performances with different sources of pollution.

The comparison of the two Plantower models confirms the manufacturer statement that these two sensors have the same performances. Zheng *et al*.^27^ obtained similar Pearson coefficients and slope when comparing 3 Plantower PMS3003 (one month in the field). The Plantower models also have a non-linear relationship with the Honeywell HPMA115S0 which may be explained by the effect of relative humidity on both sensors the latter reporting lower PM_2.5_ concentrations compared. The Alphasense OPC-N2 exhibits a different behaviour to the other sensors with three apparent modes of operation which may be linked to a combined effect of different environmental factors. The inter-comparison of the sensors by relative humidity reveals that the three makes of sensor behave differently at different relative humidity, with reported values diverging increasingly with relative humidity. This is further illustrated by the greater variability of the coefficient of correlation with the reference station for the Alphasense OPC-N2 for different levels of relative humidity. An analysis of the CV revealed a limited variation between sensor models with the Alphasense OPC-N2 presenting better values. Chen *et al*.^26^ tested nine Plantower PMS3003 sensors (36 h in the field) and found a CV < 0.4 suggesting a limited inter-sensor variability further supported by Kelly *et al*.^21^ calculation of *R*^2^ > 0.99 between the two sensors. Longer field studies with a greater number of sensor are required to precisely evaluate the inter-model variability of the Plantower models. Similarly, in studies of the Alphasense OPC-N2 against reference instruments, Crilley *et al*.^24^ found a inter-unit agreement with a CV of 0.25 ± 0.14 across 14 units while Badura *et al*.^29^ observed a CV of 0.2 across three units, values similar to those obtained in this study.

In terms of the correlation of sensor-reported PM_2.5_ concentrations with those from the nearby reference station, the two Plantower models presented very similar coefficients. During a two-day outdoor collocation of 17 Plantower PMS7003 with a TEOM, Wang *et al*.^28^ obtained *R*^2^ between 0.72–0.78 for PM_2.5_, very similar to our results. This suggests that these sensors may be suitable for reporting hourly concentration of pollution. The two Plantower models also have similar coefficients across the two sites which are 2 km apart and the time series of the hourly data shows that the six AQMs were measuring in similar surroundings. While the Alphasense OPC-N2 has lower coefficients than the Plantower models, it shows more variability across the three locations of School A which may reflect higher precision of the Alphasense OPC-N2 and a better capacity to monitor short-term localised events or be linked to external environmental factors. To confirm their precision, an extended collocation study is required.

All three sensors tended to report greater concentrations of PM_2.5_ compared to the reference station, but it should be noted that the sensors record concentrations every 1–10 s, while the TEOM records hourly averages. Moreover, the TEOM is sampling only half of the time on a 12 min cycle^46^ in a background location, so it may not detect the same temporal variations as the low-cost sensors. The sensors may be also more susceptible to local short-lived events as illustrated by the fire event and episodes at School B in August 2018. Suitability for monitoring highly spatially resolved events would be best evaluated against a reference-grade instrument with the same frequency of measurement and reporting. Sousan *et al*.^30^ also found over-reporting by the Alphasense OPC-N2 suggesting that this may be a more general property of these sensors, while Kelly *et al*.^21^ suggests this behaviour may be environmentally specific for the Plantowers sensors, although a collocation is needed to confirm that this is not simply due to higher concentrations at the AQM sites.

Wang *et al*.^47^ studied the response of Shinyei PPD42NS, Samyoung DSM501A and Sharp GP2Y1010AU0F sensors, in controlled conditions, and found that responses depended strongly on particle composition and size and that relative humidity affected the readings across a wide range of concentrations (0–1000 μg/m^3^). Thus, we expected to find variability with wind direction and months for which the sources of pollution may vary changing the particle composition and size. The results obtained here for the month of August, exhibited a clear impact of wind direction on the correlation with the background AURN station suggests the importance of particle composition and particle size, hence sources of pollution, on PM_2.5_ concentrations reported by the sensors, confirmed by a number of studies^15,19^.

Our sensors obtained Pearson coefficients >0.6 when background concentration levels were in the highest quartile which is confirmed by other studies^22^. There was no significant difference between the correlations for the two lower quartiles of background concentration <11.2 μg/m^3^. This value is similar to the Lower Limit of Detection (LLOD) advertised by the manufacturers (10 μg/m^3^). For low background concentration, the readings will likely be influenced to a greater extent by local sources of pollution. The sensors were located away from the reference station, possibly explaining the drop in correlation. To the best of our knowledge no studies have evaluated the LLOD of the Alphasense OPC-N2. For the Plantowers, Kelly *et al*. determined a LLOD of 10.5 μg/m^3^ in outdoor environments and LLOD from 1–3.22 μg/m^3^, in a laboratory. Sayahi *et al*.^32^ extended the duration of the study conducted by Kelly *et al*.^21^ from 28 datapoints to 320 days and obtained LLOD ≈6 μg/m^3^. For the Shinyei PPD42NS in laboratory conditions, Wang *et al*.^47^ reported a LLOD, of 4.28–26.9 μg/m^3^, and Austin *et al*.^20^ of 1 μg/m^3^. During a field deployment, Zikova *et al*.^48^ calculated a LLOD of 10 μg/m^3^ for 58 Syhitech DSM501A, although across only two days of deployment. We showed that the CV decreased with increasing background concentration. Zikova *et al*.^49^ used the value obtained for the LLOD to correct the data for measurements that were lower than the LLOD and obtained a better CV between sensors with the corrected data. Therefore, a higher variability between sensors is to be expected at levels of pollution below the stated LLOD.

The sensors studied presented very limited effect of relative humidity. Gao *et al*.^50^ observed a strong effect of relative humidity and temperature on sensor readings with a background of 167 μg/m^3^ while Wang *et al*.^28^ noted increased correlation between low-cost sensors and a TEOM for relative humidity 67–75% but decreased correlation for low and high relative humidity (20% and 90%), for PM concentrations 0–1,000 μg/m^3^. Conversely, Jiao *et al*.^51^ found no effect of relative humidity on the sensors readings, with average concentrations of PM_2.5_ of 10 μg/m^3^ suggesting that relative humidity may exert more of an effect at higher PM concentrations. Crilley *et al*.^24^ developed a correction factor for the Alphasense OPC-N2 for relative humidity >85%. Feinberg *et al*.^31^ reported peaks for relative humidity >90%. Jayaratne *et al*.^25^ studied the impact of relative humidity on a Plantower PMS1003 and observed that the sensor over estimated PM_2.5_ concentrations for relative humidity >80%. They suggested that the deviation observed at high relative humidity may result from the absence of heated inlet on the low-cost sensors. The sensors respond to relative humidity differently, which may be a result of testing in different environments, with different PM characteristics. In our study, the Alphasense OPC-N2 presents a higher variability with wind direction suggesting that it is more susceptible to differences in PM composition. In a recent study, Di Antonio *et al*.^33^, studied the Alphasense OPC-N2 and suggested that data should be corrected for relative humidity based on particle-size distribution rather than on PM mass and according to PM composition. However, it is unclear whether this suggestion is also applicable to other models of low-cost PM sensors given the different behaviour we observed for relative humidity between sensor model.

Temporal drift has been observed for gas sensors, with attempts made at correction^52^. Drift over time of low-cost PM sensors may result from degradation of electrical components, or dust accumulation. We saw no consistent drift over time for the two Plantowers but trend to drift was observed for the Alphasense OPC-N2. In our AQMs, the sensors facing downward may help reduce the build-up of dust inside the sensors but accumulation of particles inside the AQMs cannot be not excluded. Mukherjee *et al*.^23^ attributed drift over time for the Alphasense OPC-N2 (12 weeks in the field) to the potential build up of dust inside the sensor and on the fan.

## Conclusion

In this study, we have shown that there is general agreement in readings between the four models sensors tested, despite the differences in the way in which these sensors derive reported PM mass concentrations. The low-cost sensors show more variability at low PM concentrations and may be differentially affected by varying temperature and humidity implying the potential need for different correction methods. Despite these issues, these low-cost sensors are suitable for monitoring short-lived pollution events especially where coupled with wind data and they may provide useful information on personal exposure to PM. They may also be suitable for reporting hourly data to produce data to inform the public. The inter-model variability suggests that they should not be deployed individually, with collocation of multiple sensors also providing redundancy facilitating fault/outlier detection and ensuring full data coverage. Further work, including long-term collocation studies and laboratory testing under controlled conditions, is required to determine the precise nature and magnitude of the effects of confounding factors, leading to a better understanding of the behaviour of these low-cost sensors. Given future characterisation, low-cost sensors may be a cost-effective means to improve spatial resolution of PM monitoring in urban networks.

## Supplementary information

Supplementary Information

## Data Availability

The underlying datasets for this publication are available at 10.5281/zenodo.2605402.
