# Author Correction: Long-term field comparison of multiple low-cost particulate matter sensors in an outdoor urban environment

**DOI:** 10.1038/s41598-020-74312-5

**Published:** 2020-10-14

**Authors:** Florentin M. J. Bulot, Steven J. Johnston, Philip J. Basford, Natasha H. C. Easton, Mihaela Apetroaie-Cristea, Gavin L. Foster, Andrew K. R. Morris, Simon J. Cox, Matthew Loxham

**Affiliations:** 1 Faculty of Engineering and Physical Sciences, University of Southampton, Southampton, UK; 2 Southampton Marine and Maritime Institute, University of Southampton, Southampton, UK; 3 Faculty of Environmental and Life Sciences, University of Southampton, Southampton, UK; 4 School of Ocean and Earth Science, National Oceanography Centre, University of Southampton, Southampton, UK; 5 National Oceanography Centre, Southampton, UK; 6 Faculty of Medicine, University of Southampton, Southampton, UK; 7 National Institute for Health Research, Southampton Biomedical Research Centre, Southampton, UK; 8 Institute for Life Sciences, University of Southampton, Southampton, UK

Correction to: *Scientific Reports* https://doi.org/10.1038/s41598-019-43716-3, published online 16 May 2019.

This Article contains a typographical error in the Acknowledgements section.

“Natural Environmental Research Council grant number [NE/L002531/1]”

should read:

“Natural Environment Research Council: NE/N012070/1”.

