# Supplementary material for: Long-term field comparison of multiple low-cost particulate matter sensors in an outdoor urban environment
Source: Sci Rep. 2019 May 16;9:7497. doi: 10.1038/s41598-019-43716-3 (PMC6522472; doi:10.1038/s41598-019-43716-3)
Supplement: Supplementary file 1 — Supplementary Information [file 41598_2019_43716_MOESM1_ESM.pdf]

Supplementary Information

Long-term field comparison of multiple low-cost particulate matter sensors in an outdoor urban environment

Florentin M. J. Bulot[1,2], Steven J. Johnston[1,2,*], Philip J. Basford[1], Natasha H. C. Easton[2,3], Mihaela Apetroaie-Cristea[1], Gavin L. Foster[2,4], Andrew K. R. Morris[5], Simon J. Cox[1], and Matthew Loxham[2,6,7,8]

[1]Faculty of Engineering and Physical Sciences, University of Southampton, Southampton, UK
[2]Southampton Marine and Maritime Institute, University of Southampton, Southampton, UK
[3]Faculty of Environmental and Life Sciences, University of Southampton, Southampton, UK
[4]School of Ocean and Earth Science, National Oceanography Centre, University of Southampton, UK
[5]National Oceanography Centre, Southampton, UK
[6]Faculty of Medicine, University of Southampton, Southampton, UK
[7]National Institute for Health Research, Southampton Biomedical Research Centre, Southampton, UK
[8]Institute for Life Sciences, University of Southampton, Southampton, UK
[*]sjj698@zepler.org

1 Description of the Air Quality Monitors

The Air Quality Monitor (AQM) is a modular air quality sensor platform which enables the deployment of multiple sensors, with data connectivity for remote administration and cloud based data storage. The enclosure measures 360 mm×200 mm×160 mm (H×W×D) and is weather-proof. The sensors are positioned with inlets facing downwards and the air flows through the enclosure through a grid of 8 mm diameter holes at the base of the enclosure and are protected from debris and biological material by a 3 mm diameter mesh. AQMs are controlled by a Raspberry Pi and are powered by Power over Ethernet. Each AQM includes the four different models of Particulate Matter (PM) sensors and a sensor for temperature and humidity (DHT22[1]).

2 Supplementary Figures

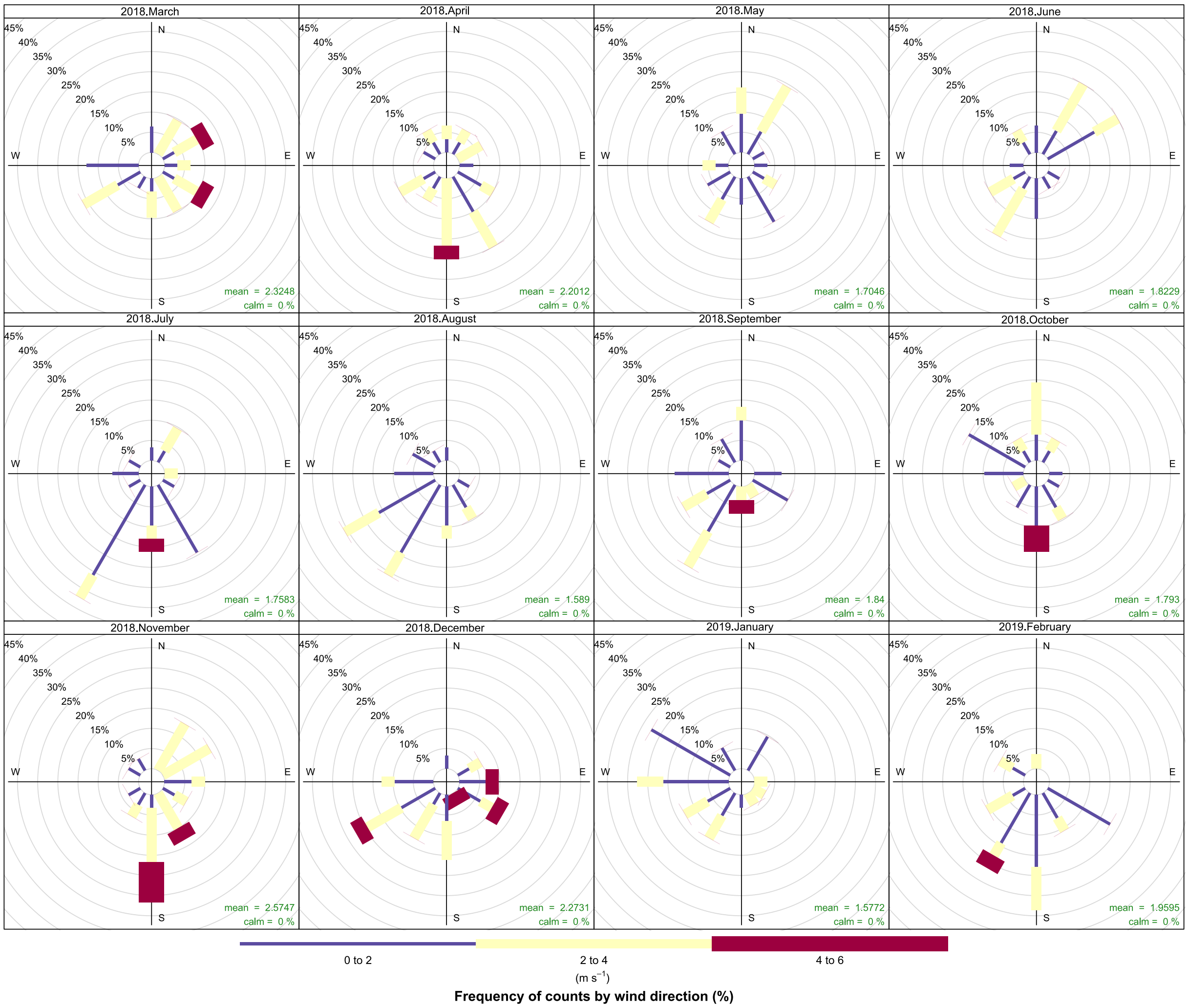

Figure S1. Wind roses per month during the study.

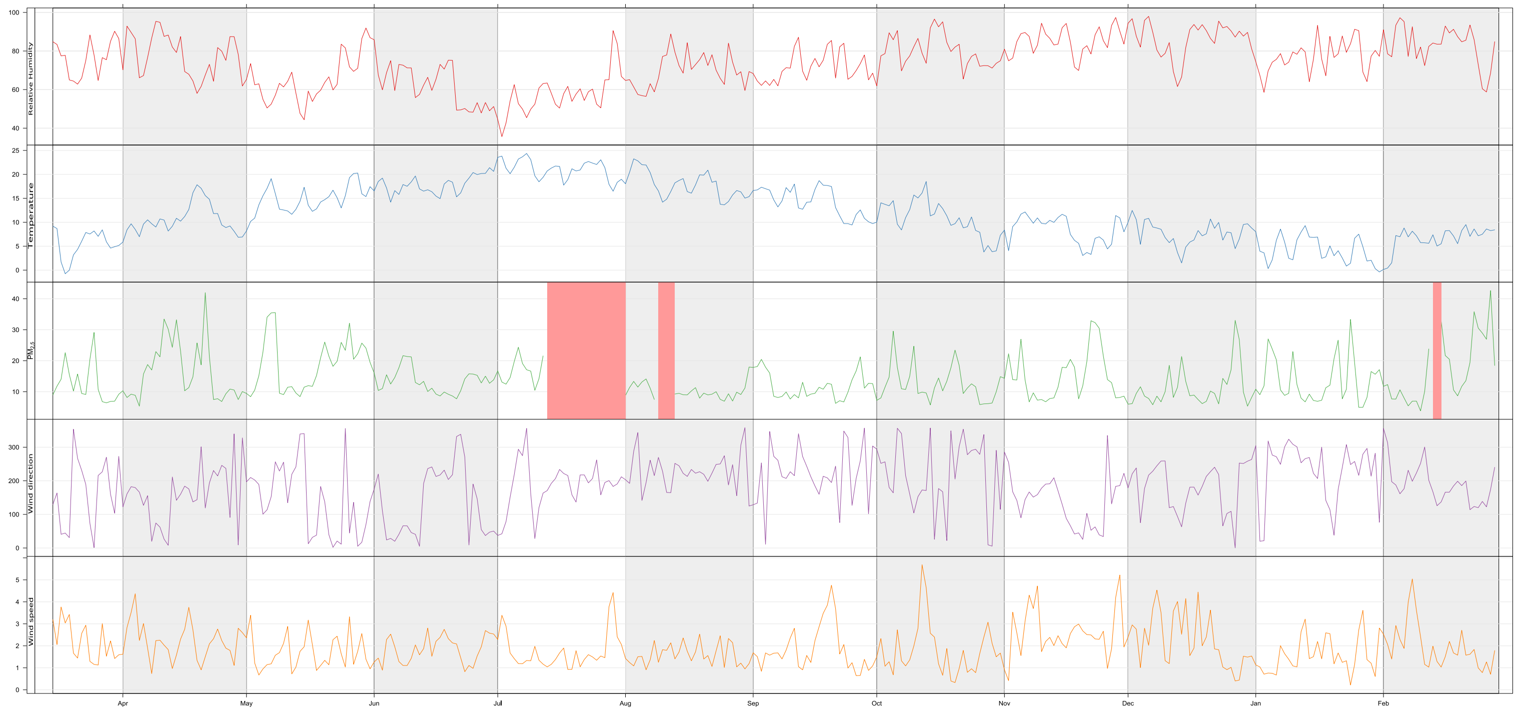

Figure S2. Time series of the daily averages for relative humidity, temperature, background concentration of $PM_{2.5}$ and wind direction and direction from 13/03/18 until 28/02/19 in Southampton, UK. Red shaded area represent periods of missing data for the reference station. $PM_{2.5}$ concentrations generally followed a daily repeating pattern with peaks around 07:00 and 20:00 and a weekly increasing during the week until Saturday to decrease on Sunday.

The mean $PM_{2.5}$ concentration registered by the Automatic Urban and Rural Network (AURN) station was 14.1 $\mu g/m^3$. $PM_{2.5}$ concentration were generally higher during April 2018, May 2018, and February 2019 with respectively 10, 14 and 9 days $>20\,\mu g/m^3$ including 4 days $>30\,\mu g/m^3$ each and respectively 21, 25 and 18 days $>10\,\mu g/m^3$. July 2018 also reported $PM_{2.5}$ concentrations similar to the three above mentioned months but with only two days $>20\,\mu g/m^3$ and 12 days $>10\,\mu g/m^3$. June 2018 also registered 24 days $>10\,\mu g/m^3$. The other months each registered <7 days with concentrations of $PM_{2.5}$ $>20\,\mu g/m^3$ and between 7-13 days $>10\,\mu g/m^3$. August 2018 was the month with the lowest mean concentration of $PM_{2.5}$ followed by September 2018 and December 2018.

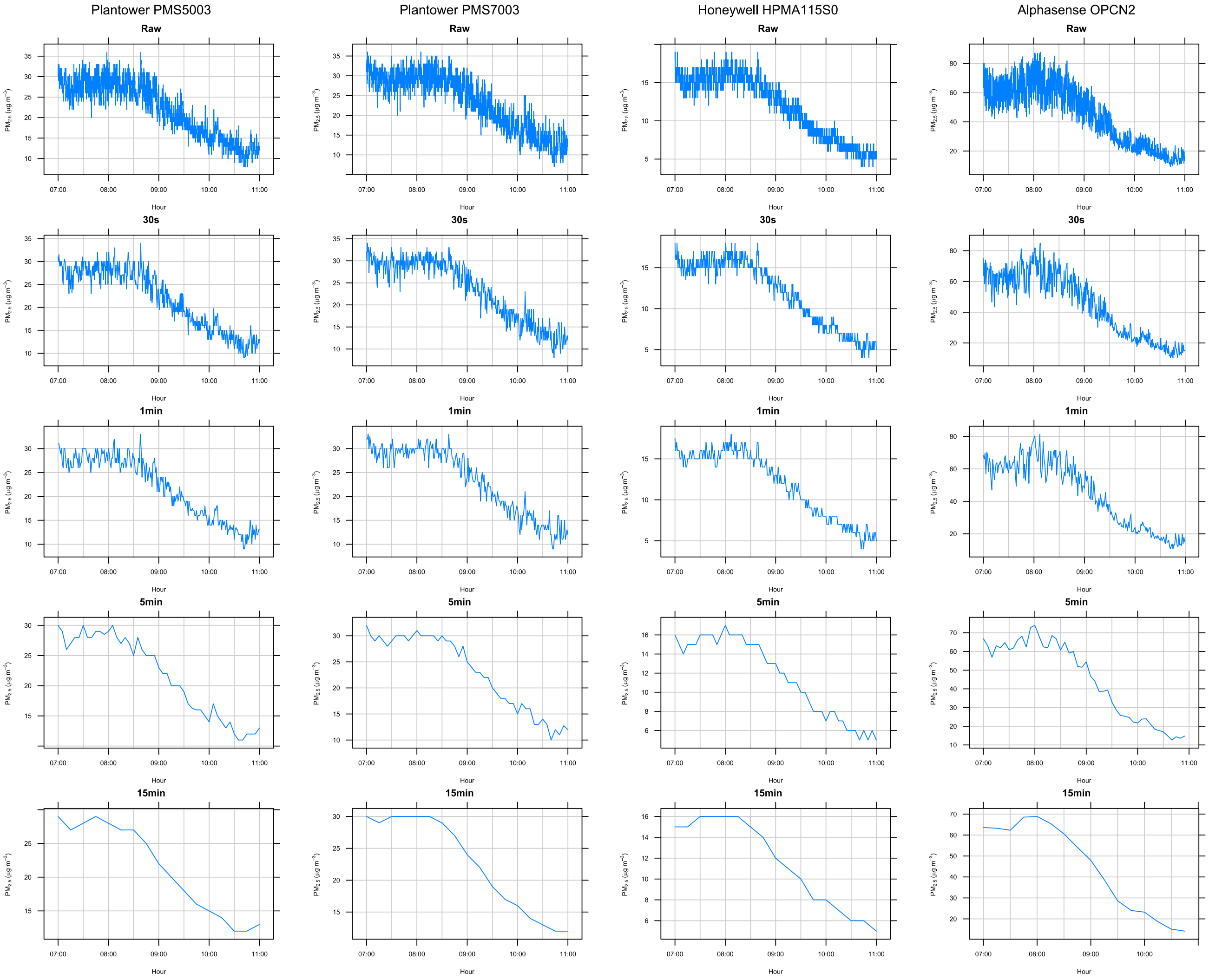

Figure S3. Comparison of different time average windows for the Plantower PMS5003, Plantower PMS7003, Alphasense OPC-N2 and Honeywell HPMA115S0 for AQM A.1 on 09/04/18 from 07:00 to 11:00.

AQM	Date	Sensor	Mean (μg/m^3)
AQM A.3	16/09/18 to 23/09/18	Plantower 5003	4.2
		Plantower 7003	54.0
		Alphasense OPCN2	7.0
	23/09/18 to 30/09/18	Plantower 5003	8.7
		Plantower 7003	93.6
		Alphasense OPCN2	4.8
	30/09/18 to 07/10/18	Plantower 5003	7.9
		Plantower 7003	8.8
		Alphasense OPCN2	8.9
AQM B.1	30/09/18 to 07/10/18	Plantower 5003	877.0
		Plantower 7003	6.1
	07/10/18 to 14/10/18	Plantower 5003	3358.4
		Plantower 7003	7.2
	14/10/18 to 21/10/18	Plantower 5003	1145.9
		Plantower 7003	9.0

Table S1. Weekly mean concentrations for sensors in AQM A.1 and AQM B.3 illustrating data quality check process category 3.

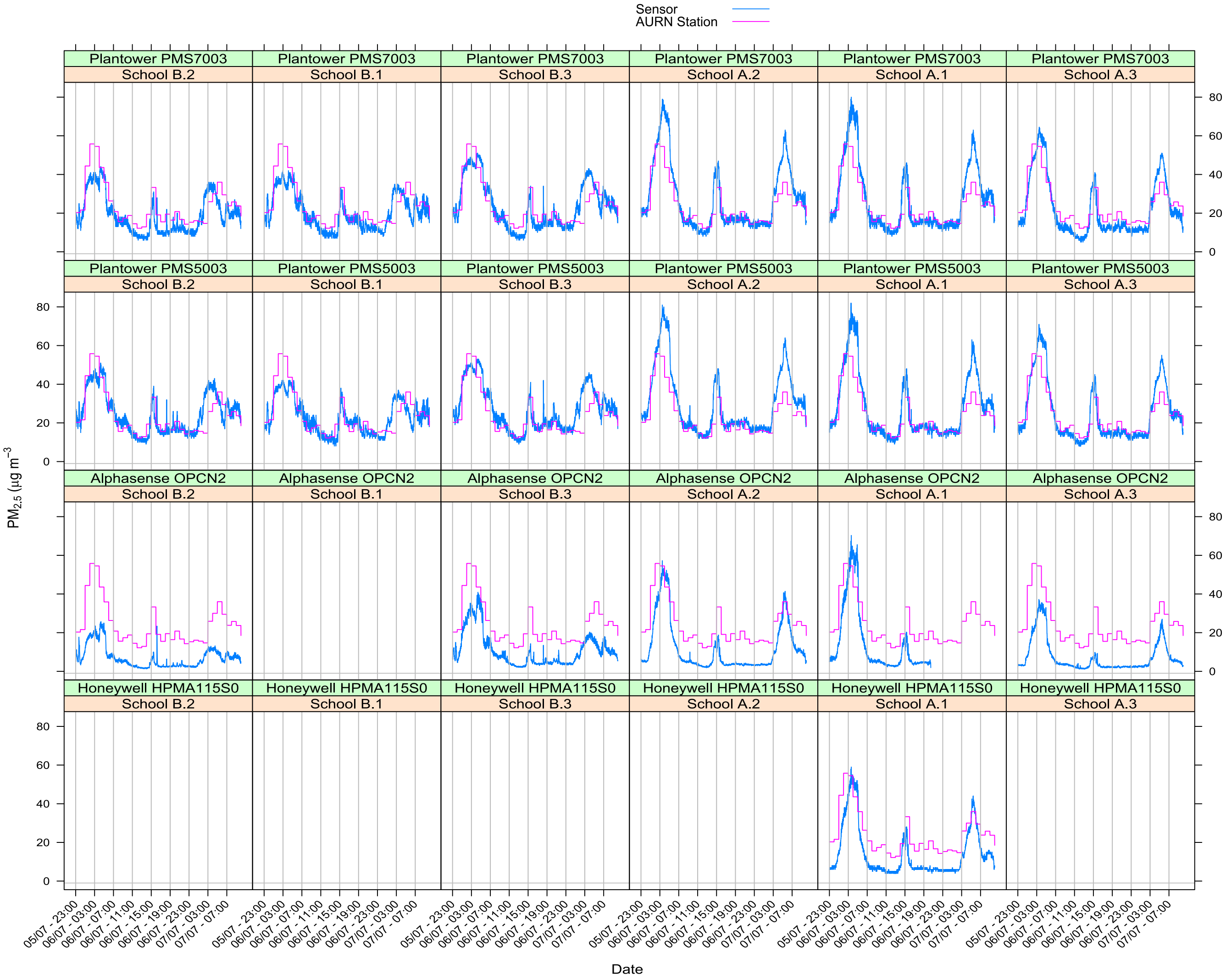

Figure S4. Comparison of the hourly measurements from the AURN station and the 1 min measurements from all the sensors between 05/07/18 and 07/07/18 11:00. The 3 spikes observed are an example of data falling into the data quality check category 4.

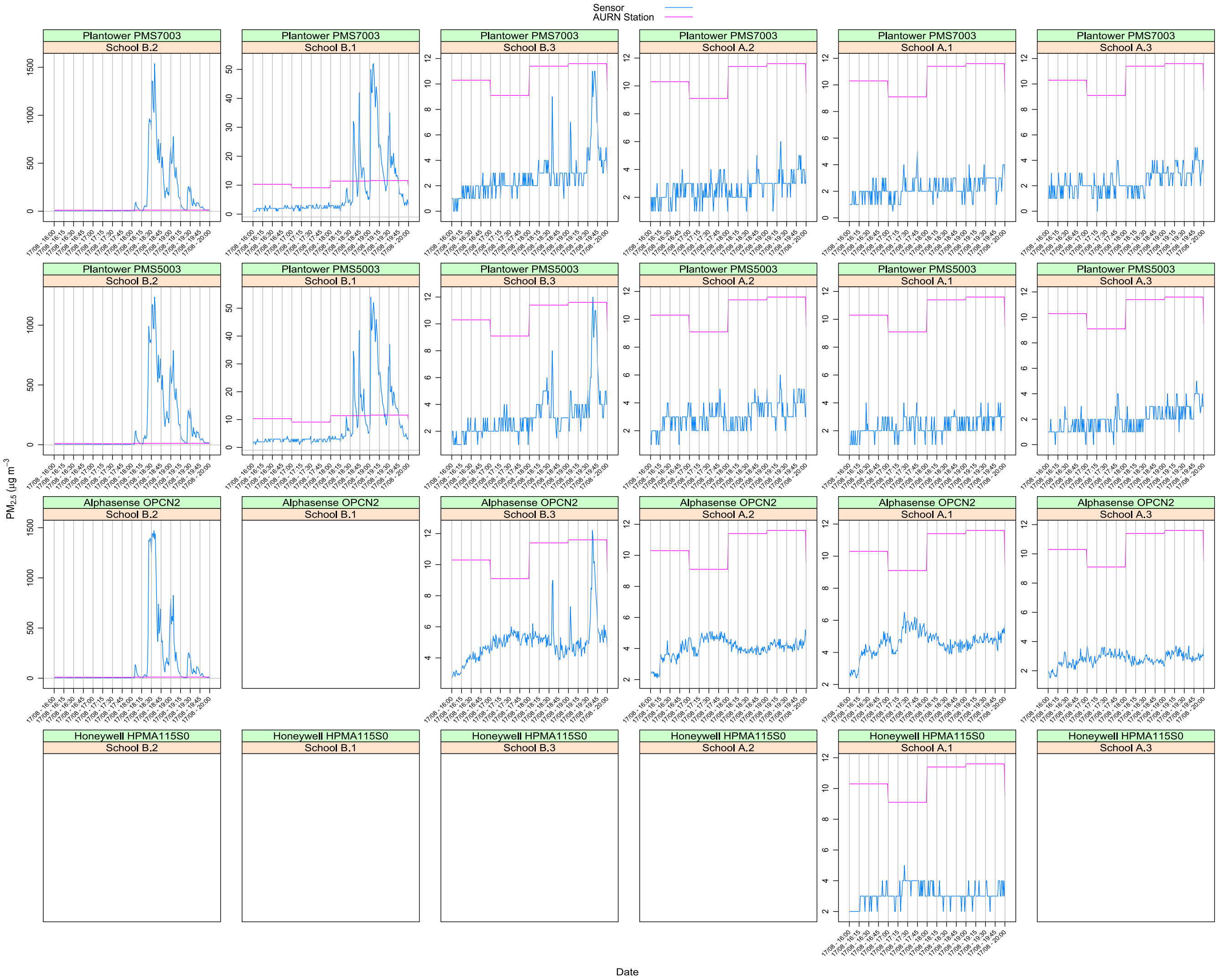

Figure S5. Comparison of the hourly measurements from the AURN station and the 1 min measurements from all the sensors between 17/08/18 16:00 and 17/08/18 20:00. The spikes observed for the 3 sensors in Schools B.2 and B.1 are an example of data falling into the data quality check category 5.

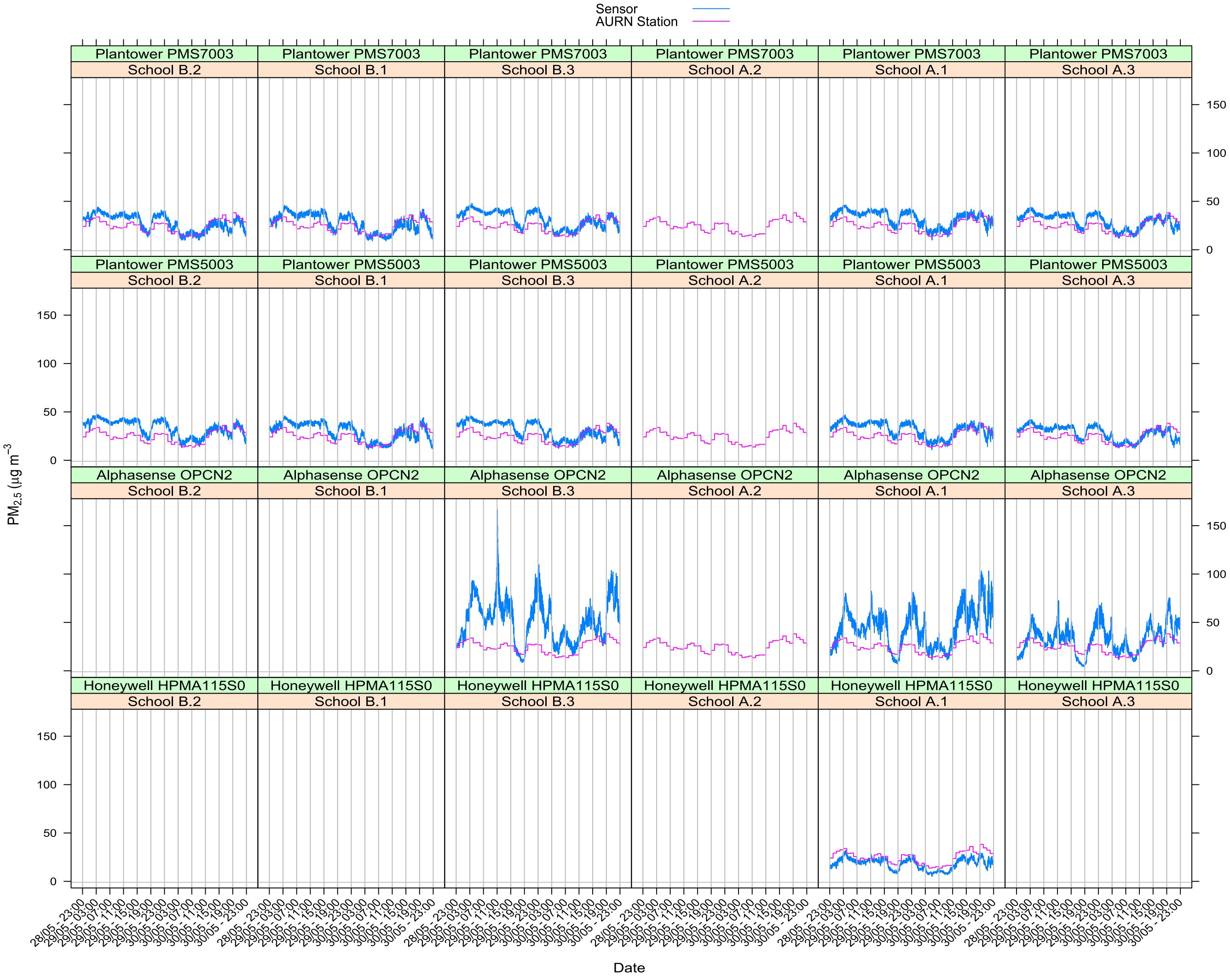

Figure S6. Comparison of the hourly measurements from the AURN station and the 1 min measurements from all the sensors between 28/05/18 and 31/05/18. The spikes observed for the Alphasense OPC-N2 at Schools B.3, A.1 and A.3 are an example of data falling into the data quality check category 6.

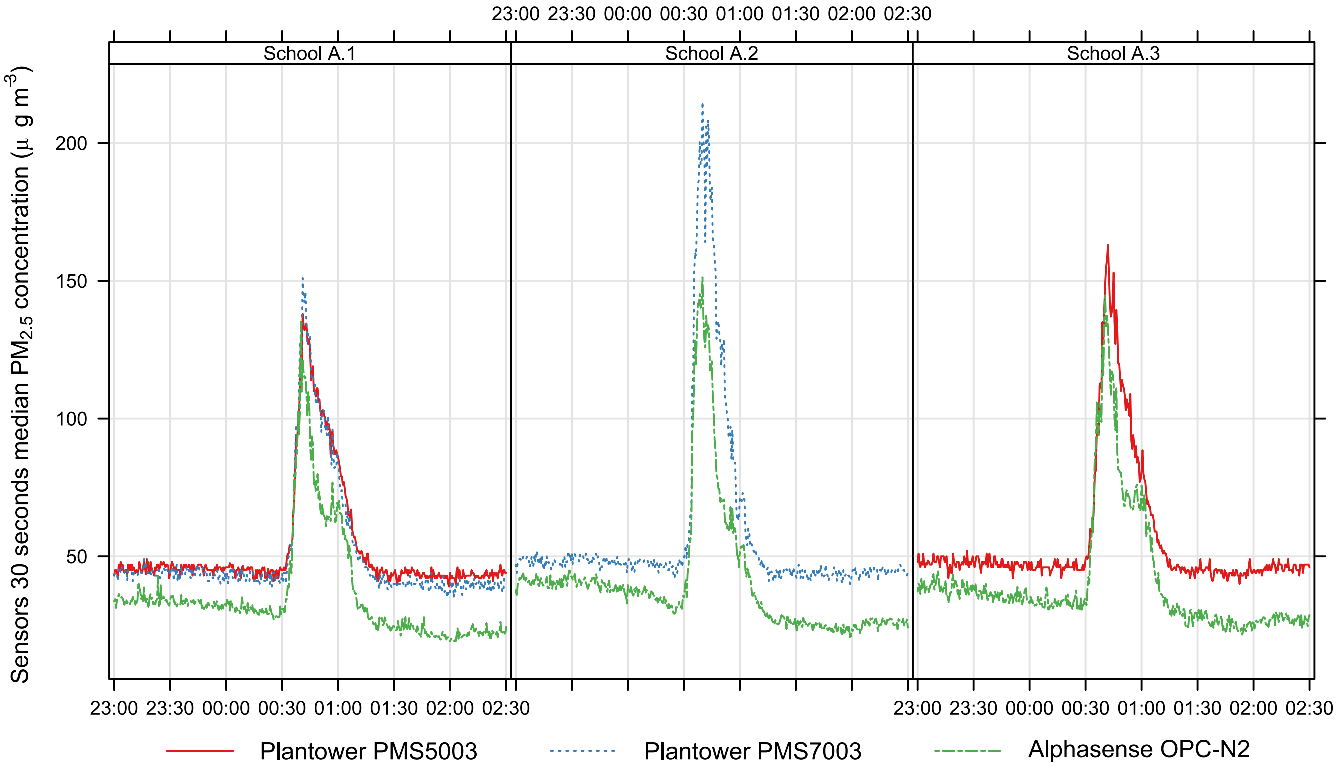

Figure S7. Changes in reported $PM_{2.5}$ concentrations during a nearby fire. Graphs show variation in reported $PM_{2.5}$ concentration at the time of a large fire in Southampton on the 24/02/2018[2], reported between 00.27 and 02.28 East-North-East of School A and of the reference station while the wind was blowing from East-North-East. The x-axis starts on the 23/02/18 at 23.00 and ends on the 24/02/18 at 02.30.

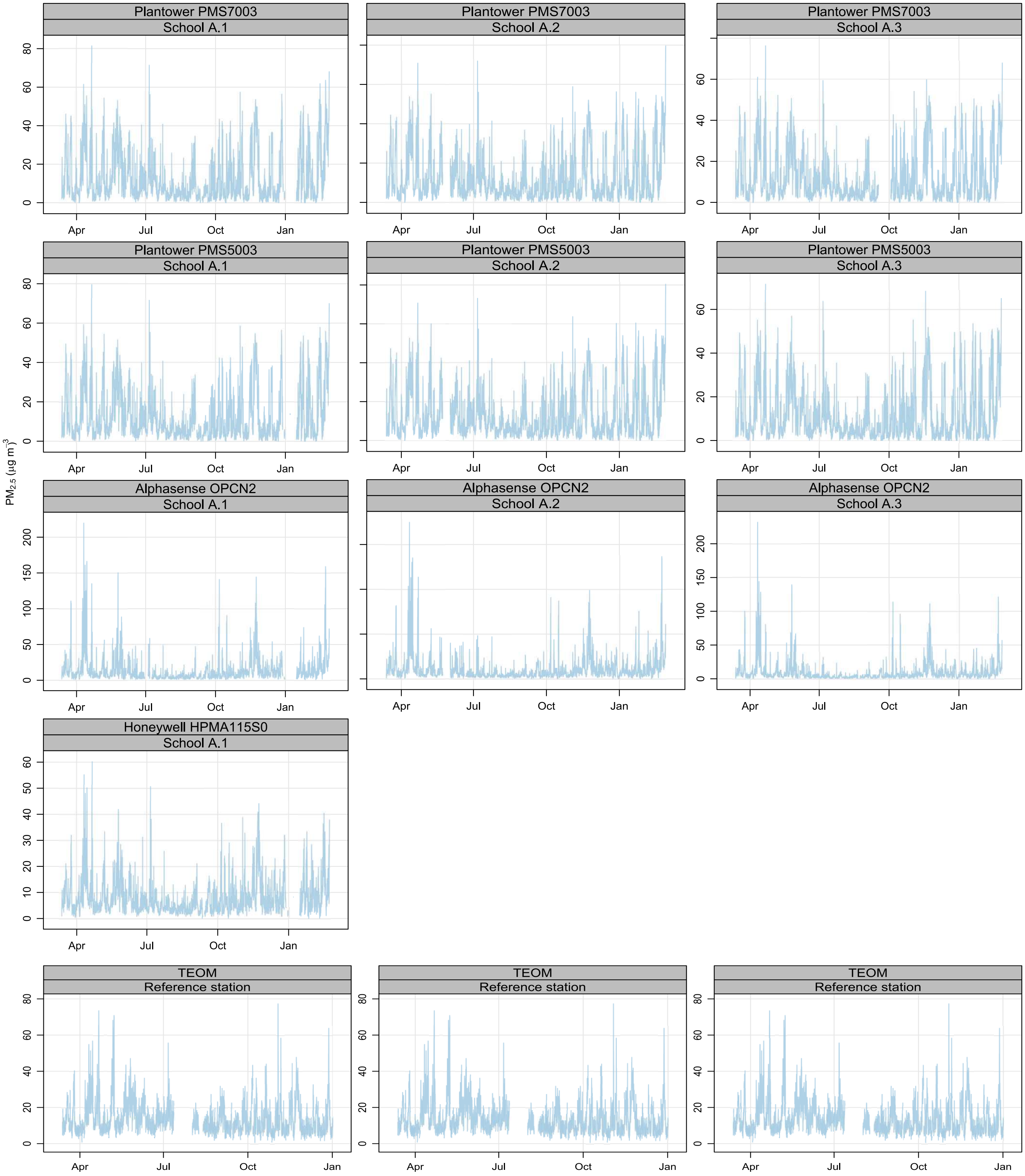

Figure S8. Time series of the hourly mean $PM_{2.5}$ concentration reported by the sensors deployed in School A and comparison with the concentration reported by the reference station. The readings of the reference station are included three times at the bottom for comparison.

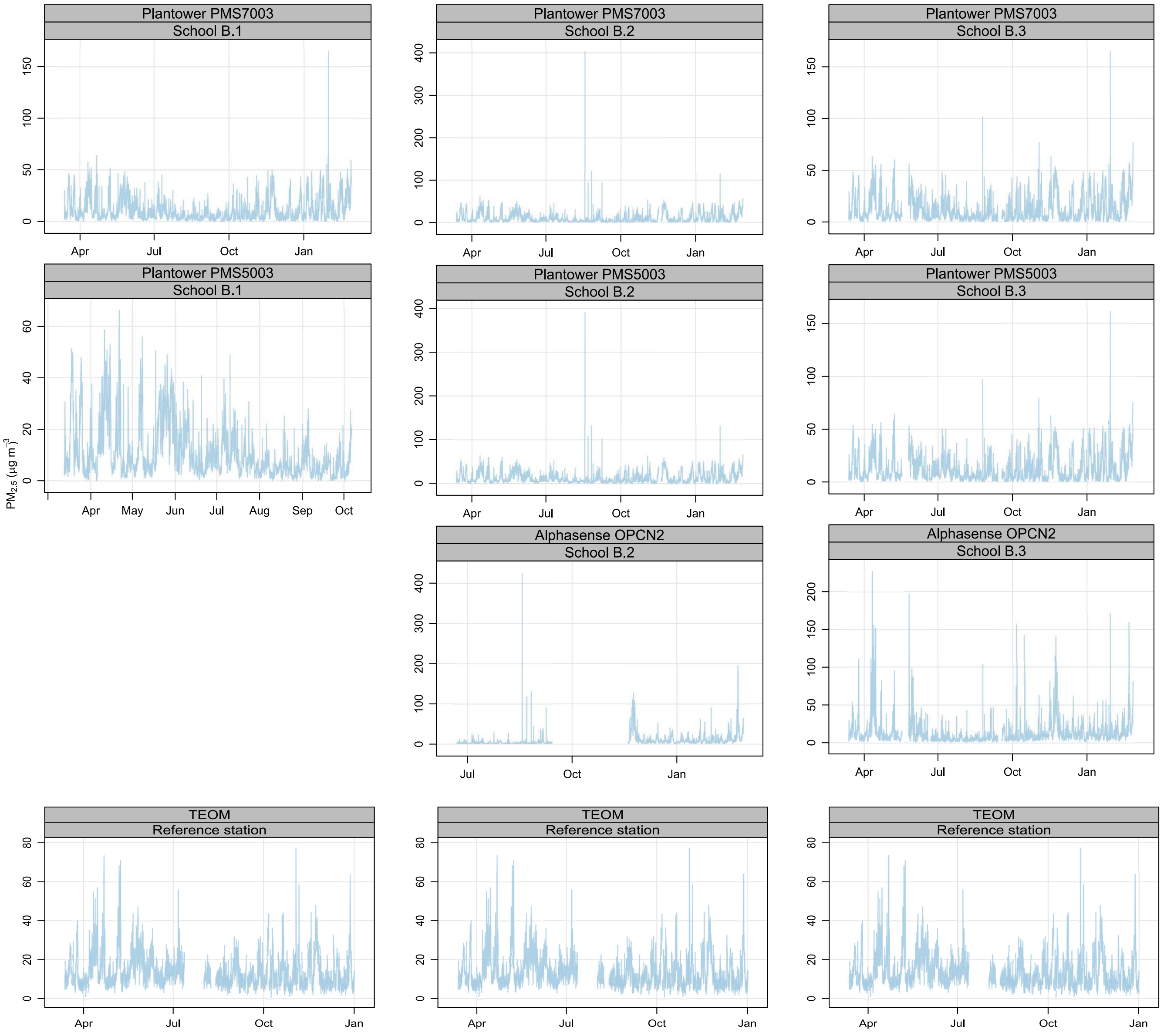

Figure S9. Time series of the hourly mean $PM_{2.5}$ concentrations reported by the sensors deployed in School B and comparison with the concentration reported by the reference station. The readings of the reference station are included three times at the bottom for comparison.

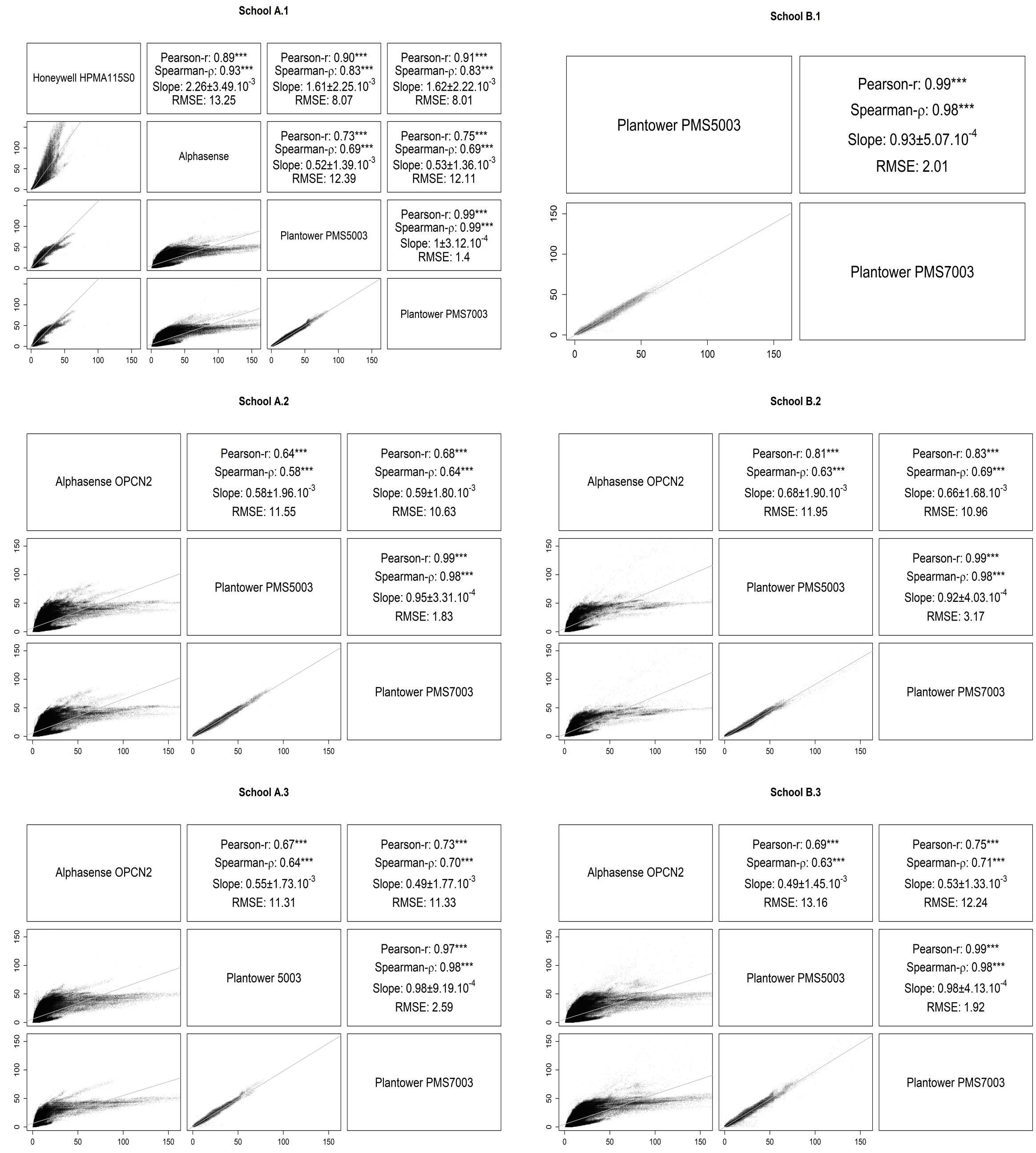

Figure S10. Correlation of the concentration of $PM_{2.5}$ in $\mu g/m^3$ reported by different PM sensor models at each AQM site. Graphs show reported $PM_{2.5}$ concentrations from the Plantower PMS5003, Plantower PMS7003, Alphasense OPC-N2 and Honeywell HPMA115S0 sensors, per location. The x-axis corresponds to the sensor named above the graph and the y-axis correspond to the sensor named to the right of the graph. The upper 0.00001 % of the datapoints are not displayed. Slope is reported ± 2 standard error (***: $p < 2 \times 10^{-16}$)

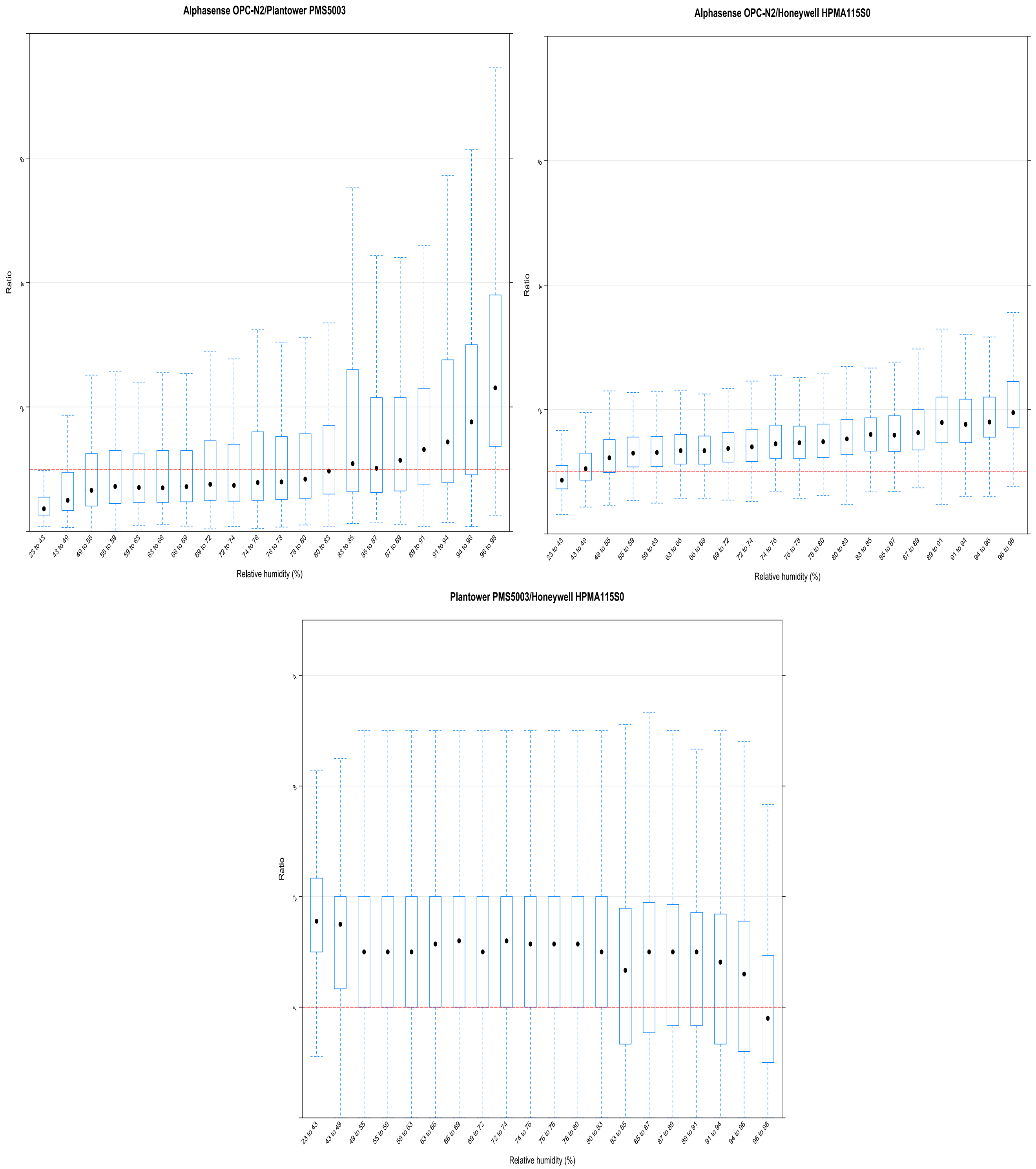

Figure S11. Ratios of the 1 min $PM_{2.5}$ concentrations reported between the Alphasense OPC-N2, the Plantower PMS5003 and the Honeywell HPMA115S0 for different levels of relative humidity. Each box represents the median, the 25th and 75th quartiles, the maximum and the minimum of the ratios observed.

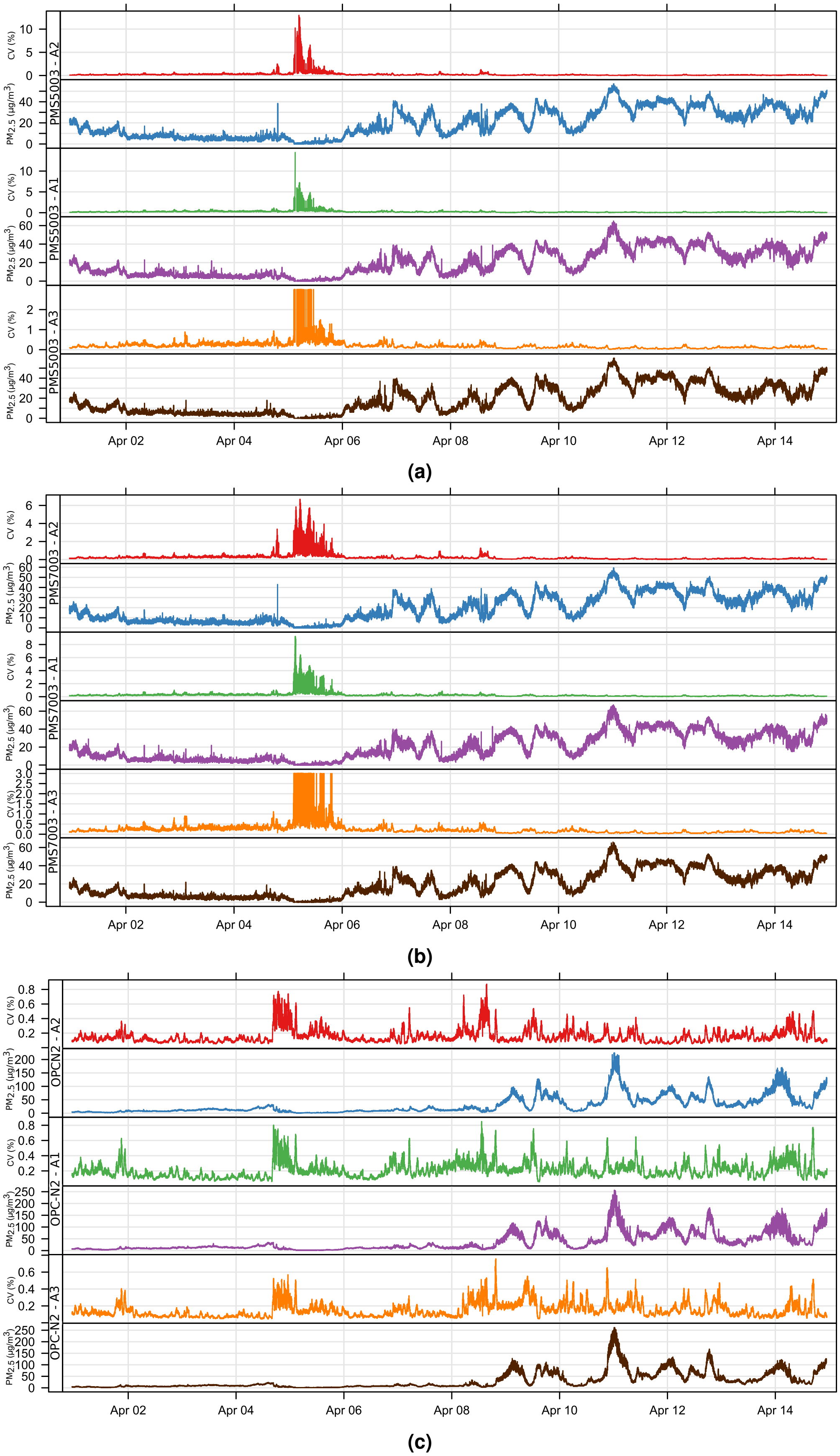

Figure S12. Coefficient of variation (CV) and $PM_{2.5}$ concentration measured by (a) three Plantower PMS5003, (b) three Plantower PMS7003 and (c) three Alphasense OPC-N2 at School A between 1st April until 15th April. Similar behaviour was observed for the other sensors at the other locations.

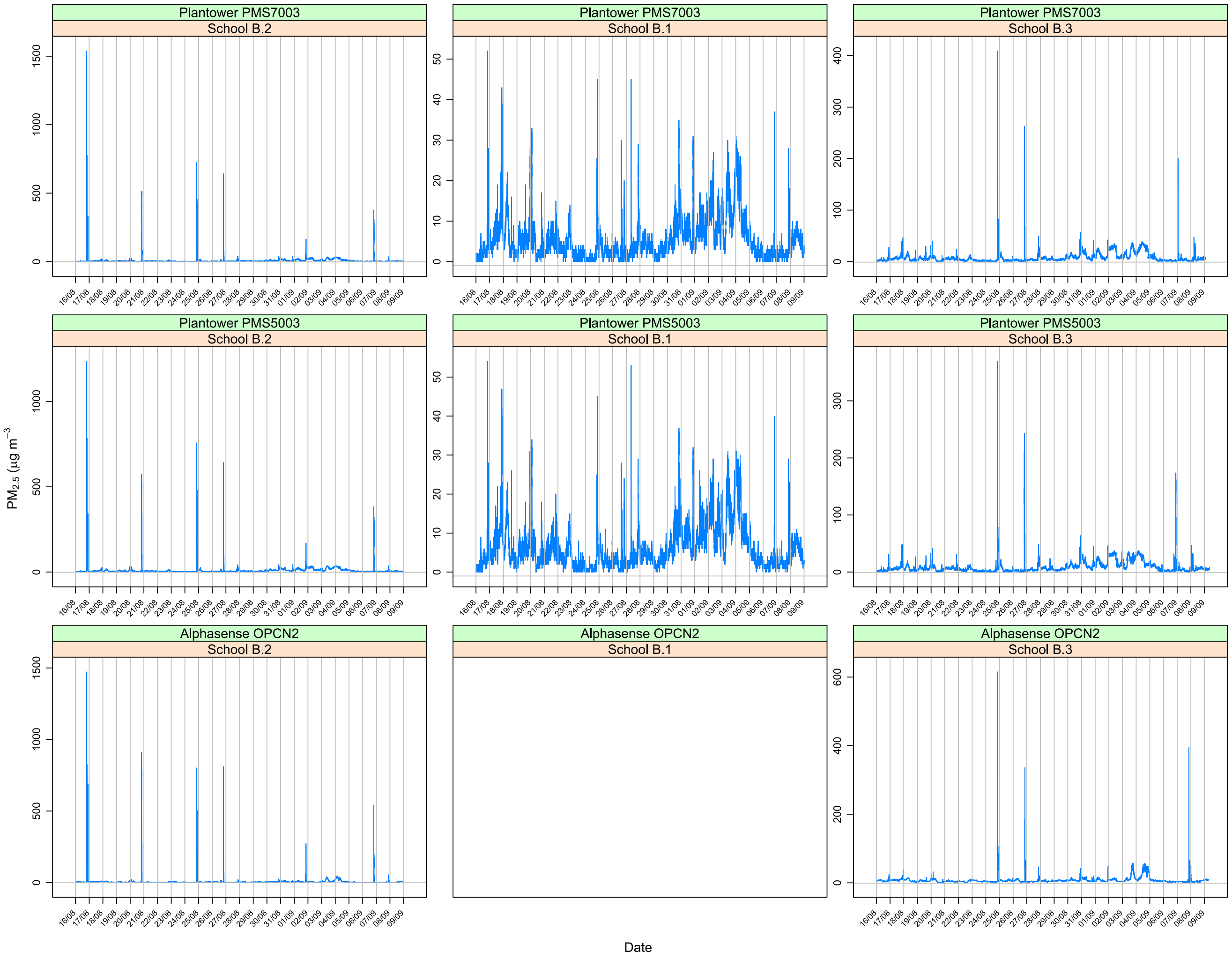

Figure S13. Spikes registered in School B between 18/08/18 and 10/09/18.

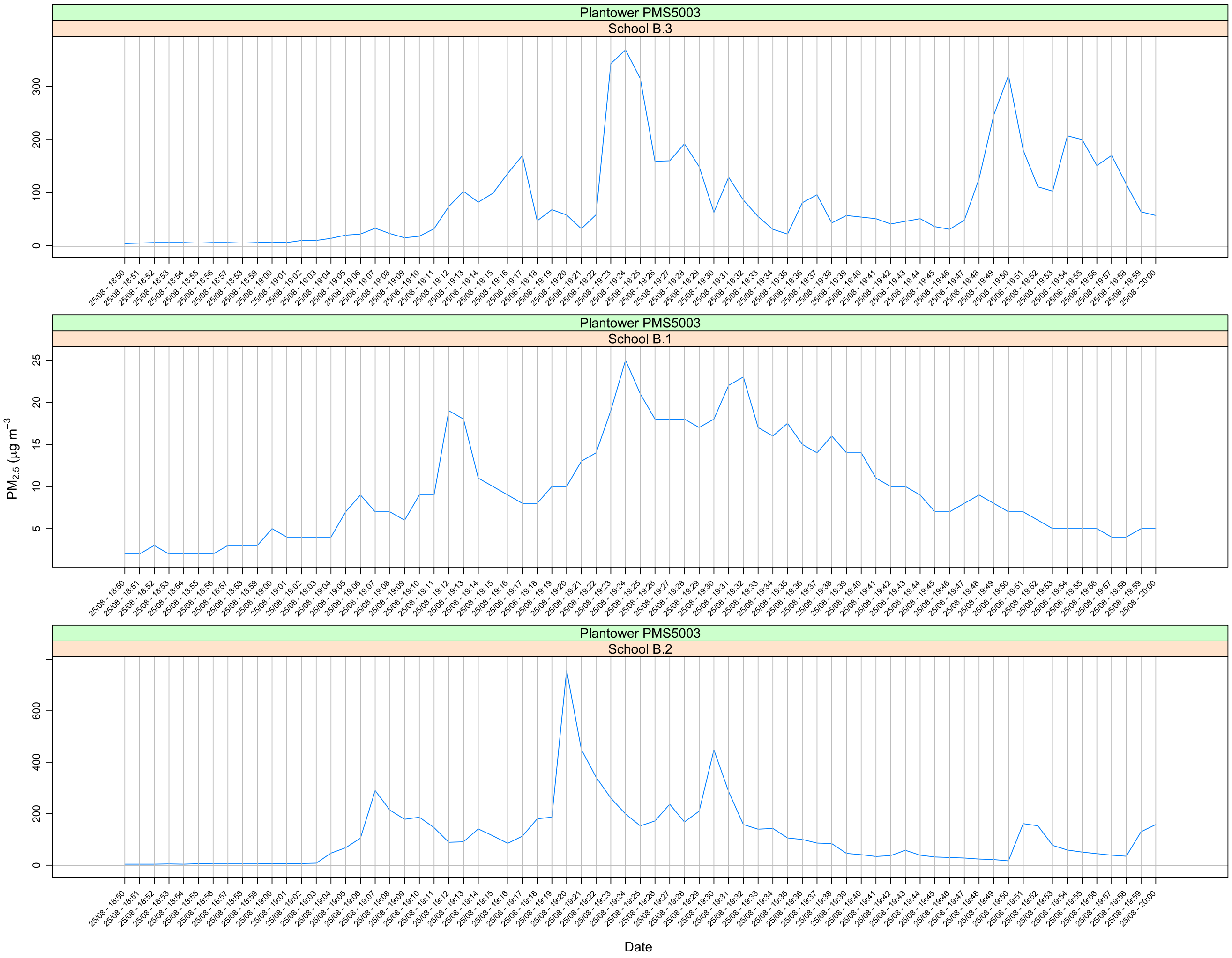

Figure S14. Spikes registered in School B between 25/08/18 18:50 and 25/08/18 20:00. Wind speed was <0.8 m/s and wind direction was WSW.

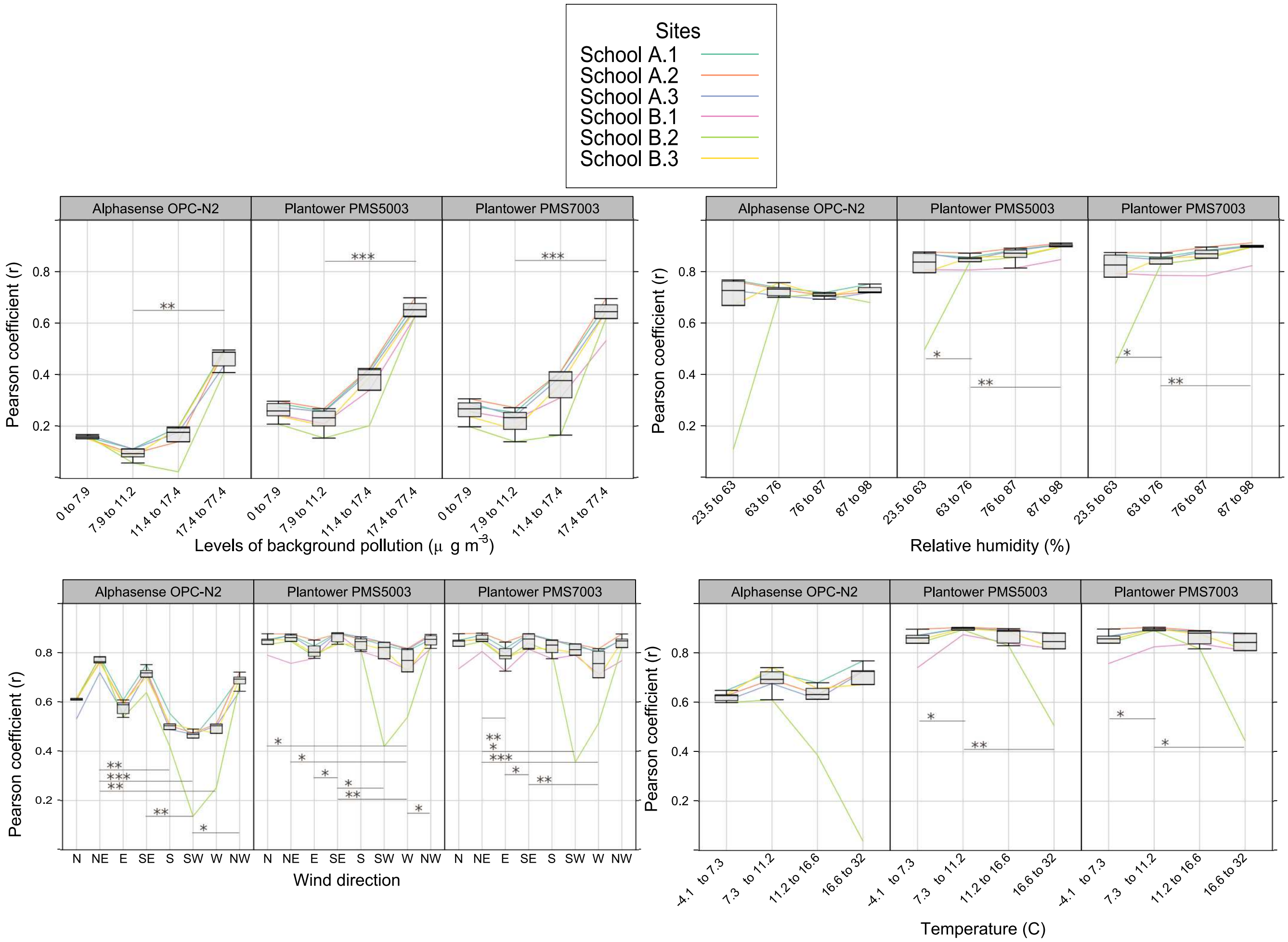

Figure S15. Effect of pollution and climate factors on veracity of low cost sensor readings. Graphs show variation in Pearson coefficient between the 3 sensor models and the background AURN reference station per site, including the month of August, with the (1) background concentration, (2) relative humidity, (3) wind direction and (4) temperature. Each box represents the median, the 25% and 75% quartiles, the maximum and the minimum of the Pearson coefficient for the locations considered (n=4 for the Alphasense OPC-N2 and n=6 the Plantowers). Data were analysed using a Friedman analysis of variance (ANOVA) with Dunn's post-hoc test for pairwise comparison and the bars represent categories which have a statistically significant difference, *$p<0.05$, ** $p<0.01$,*** $p<0.001$,**** $p<0.0001$.

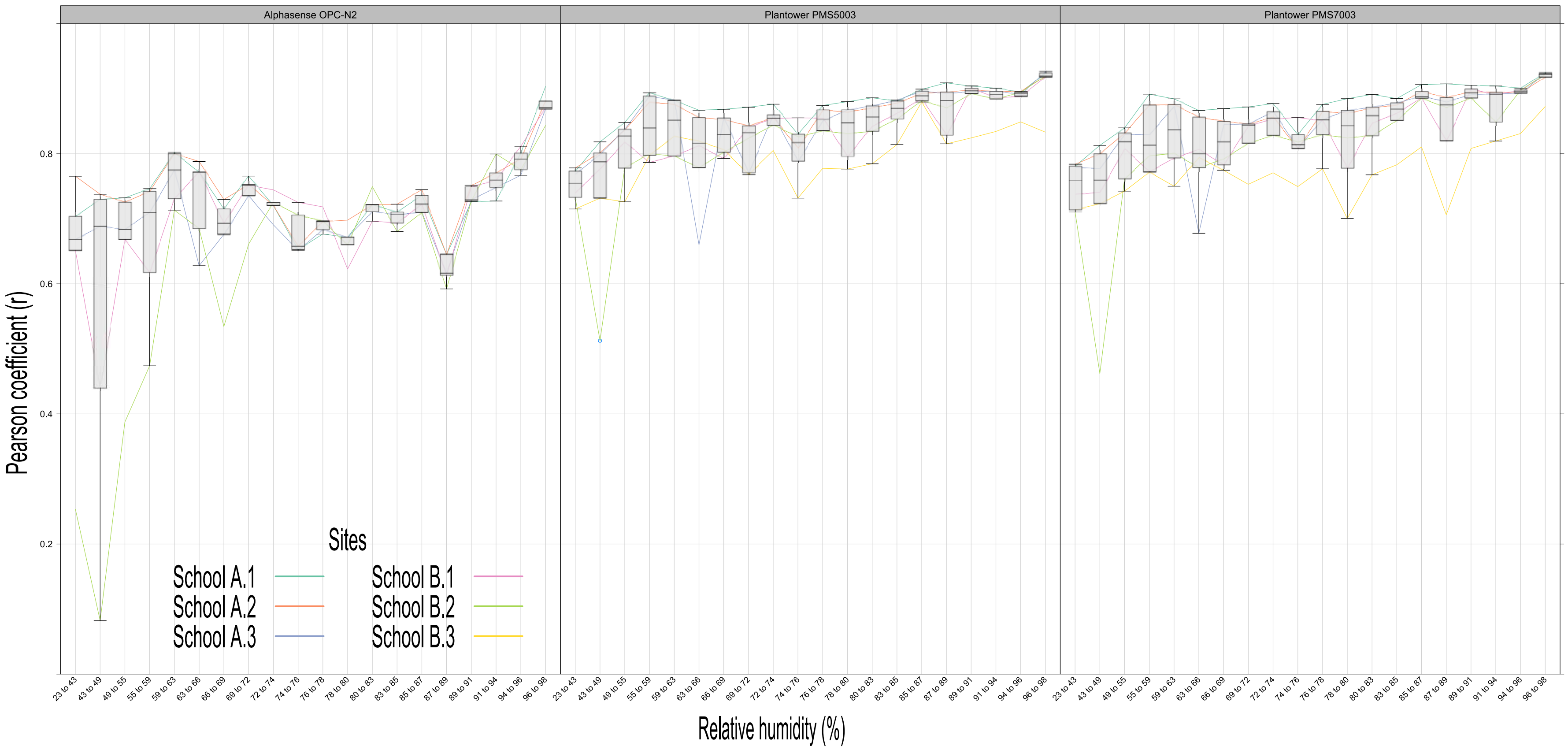

Figure S16. Effect of pollution and climate factors on veracity of low cost sensor readings. Graphs show variation in Pearson coefficient between the 3 sensors and the background AURN reference station per site according to the relative humidity. Each box represents the median, the 25 % and 75 % quartiles, the maximum and the minimum of the Pearson coefficient for the locations considered (n=4 for the Alphasense OPC-N2 and n=6 the Plantowers).

References

1. Aosong (Guangzhou) Electronics Co. L. Digital-output relative humidity and temperature sensor module am2303. *DHT22* https://cdn-shop.adafruit.com/datasheets/DHT22.pdf.
2. Hampshire fire and rescue service: incidents of interest. *Hantsfire* https://www.hantsfire.gov.uk/incidents-news-and-events/incidents-of-interest/.
